# Synthesis of C6-modified mannose 1-phosphates and evaluation of derived sugar nucleotides against GDP-mannose dehydrogenase

**DOI:** 10.3762/bjoc.18.142

**Published:** 2022-09-30

**Authors:** Sanaz Ahmadipour, Alice J C Wahart, Jonathan P Dolan, Laura Beswick, Chris S Hawes, Robert A Field, Gavin J Miller

**Affiliations:** 1 Department of Chemistry & Manchester Institute of Biotechnology, The University of Manchester, 131 Princess Street, Manchester, M1 7DN, UK; 2 Lennard-Jones Laboratory, School of Chemical and Physical Sciences, Keele University, Keele, Staffordshire, ST5 5BG, UK; 3 Centre for Glycosciences, Keele University, Keele, Staffordshire, ST5 5BG, UK

**Keywords:** alginate, chemical probe, enzymatic synthesis, GDP-mannose dehydrogenase, sugar nucleotide

## Abstract

Sufferers of cystic fibrosis are at significant risk of contracting chronic bacterial lung infections. The dominant pathogen in these cases is mucoid *Pseudomonas aeruginosa.* Such infections are characterised by overproduction of the exopolysaccharide alginate. We present herein the design and chemoenzymatic synthesis of sugar nucleotide tools to probe a critical enzyme within alginate biosynthesis, GDP-mannose dehydrogenase (GMD). We first synthesise C6-modified glycosyl 1-phosphates, incorporating 6-amino, 6-chloro and 6-sulfhydryl groups, followed by their evaluation as substrates for enzymatic pyrophosphorylative coupling. The development of this methodology enables access to GDP 6-chloro-6-deoxy-ᴅ-mannose and its evaluation against GMD.

## Introduction

The opportunistic Gram-negative pathogen, *Pseudomonas aeruginosa* (PA), becomes the dominant pathogen in patients suffering from cystic fibrosis (CF) and causes a chronic respiratory infection that infects more than 80% of those affected [1]. PA strains colonising the respiratory tract in CF undergo a phenomenon known as mucoid conversion, a phenotype characterised by the secretion of copious amounts of the carbohydrate exopolysaccharide alginate. The manifestation of alginate overproduction contributes to the PA biofilm environment and the resultant and deleterious bacterial resistance to current antibiotic treatments [2]. Alginate production is therefore established as a major virulence factor within PA respiratory tract infections for CF sufferers, and interventions that could halt its production would be of significant value.

**Figure 1:**
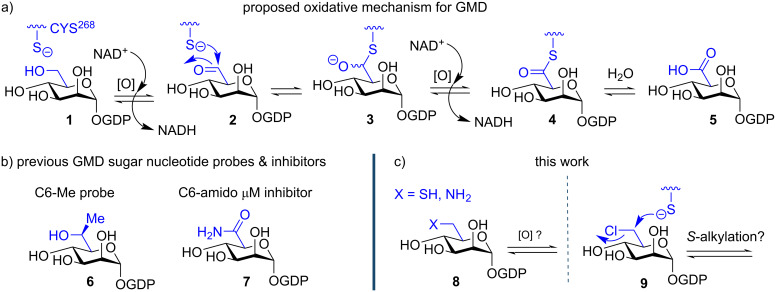
a) Proposed oxidative pathway for provision of GDP-ManA **5** from GDP-Man **1**, C6 stereochemistry of **3** is unknown; b) previously disclosed C6–Me and C6–amido structure-to-function tools for GMD; c) C6-modified GDP-Mans of type **8** and **9**, targeted in this work.

Within the biosynthetic assembly of alginate there is a critical dependence upon the provision of one sugar nucleotide building block, GDP-mannuronic acid (GDP-ManA, **5**). This material is sourced from the cytosolic metabolic pool, starting from fructose 6-phosphate and its synthesis ultimately arrives at the limiting step in alginate precursor biosynthesis, the action of GDP-mannose dehydrogenase (GMD), which oxidises GDP-mannose **1** to **5** (Figure 1a). GMD oxidation is suggested to have four discrete steps, first oxidising C6 to an aldehyde **2** followed by substrate attachment through Cys^268^ to form thiohemiacetal **3**. A second oxidation reveals thioester **4** which, following hydrolysis, releases the product **5**. As GMD does not exist in humans, strategies that could prevent its mechanism of action could open a pathway for new and selective inhibitors to disrupt bacterial alginate production [3].

We recently disclosed the first series of targeted sugar nucleotide probes for GMD (Figure 1b) [4–6]. A C6-methyl analogue **6** was oxidised by GMD with direct evidence for a ketone product obtained. Most recently, C6-amide sugar nucleotide **7** was shown to be a micromolar inhibitor of GMD (IC_50_ = 112 μM). Access to these chemical tools was established using a chemoenzymatic approach, whereby bespoke structural modifications were made to the mannose component, delivering an appropriate glycosyl 1-phosphate, followed by pyrophosphorylative enzymatic coupling to complete the sugar nucleotide [7–8].

With this capability in place, we wished to explore the synthesis of further tools, targeting the active site cysteine residue (Figure 1c). We envisaged that access to C6–amino or C6–sulfhydryl species of type **8** would offer prospect to establish active site thiohemiaminal (amine to imine oxidation) or disulfide formation, respectively. Additionally, C6–Cl derivative **9** could probe cysteine alkylation. Reported herein is our exploration of this synthesis and the evaluation of GDP 6-chloro-6-deoxy-ᴅ-mannose **18** against GMD.

## Results and Discussion

### Chemical synthesis of 6-chloro-6-deoxy- and 6-amino-6-deoxy-mannose 1-phosphates

**Scheme 1:**
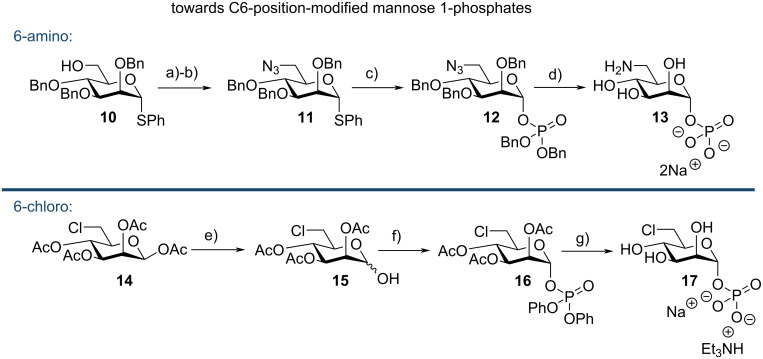
Syntheses of C6-modified mannose 1-phosphates **13** and **17**. Conditions a) PPh_3_, CBr_4_, DCM, rt, 75%; b) NaN_3_, DMF, 75 °C, 64%; c) HO(O)P(OBn)_2_, NIS, AgOTf, DCM, rt, 65%; d) Pd/C, Pd(OH)_2_, H_2_, HCl, EtOH, THF, 24 h, 90%; e) NH_4_OAc, DMF, 30 °C, 80%; f) *n*-BuLi, Cl(O)P(OPh)_2_, THF, −78 °C to rt, 58%; g) i) PtO_2_, H_2_, EtOH, ii) Et_3_N, H_2_O, MeOH, 99%, 2 steps.

We first required access to appropriate glycosyl 1-phosphates (as putative substrates for enzymatic pyrophosphorylative coupling) to then access the sugar nucleotide GMD probes of type **8** and **9** (Figure 1c). Accordingly, syntheses of C6-modified mannose 1-phosphates **13** and **17** were developed (Scheme 1).

The synthesis of 6-amino-6-deoxymannose 1-phosphate **13** started from protected thioglycoside **10** [6]. A two-step modification using Appel halogenation followed by nucleophilic substitution with azide furnished **11**. Conversion of the intermediate C6–bromide to **11** was confirmed by ^13^C NMR, with C6 shifting downfield from δ_C_ 33.4 ppm to 51.6 ppm. Following this, dibenzyl phosphate was glycosylated with **11** using NIS/AgOTf activation of the thioglycoside, which proceeded in good yield (65%) to deliver **12**. A final global deprotection with concomitant azide reduction was completed using hydrogen and Pd/C and Pd(OH)_2_/C catalysts, affording **13** as the disodium salt in 90% yield, after anion exchange chromatography.

Synthesis of 6-deoxy-6-thio-ᴅ-mannose 1-phosphate (**18**) was completed from **14** via C6 substitution with thioacetate and MacDonald phosphorylation [9]. Details of this synthesis were reported by us previously [10]. In this work, we attempted to use these optimised MacDonald conditions to directly access **17** from **14**, but the reaction exhibited extensive degradation upon multiple attempts.

**Figure 2:**
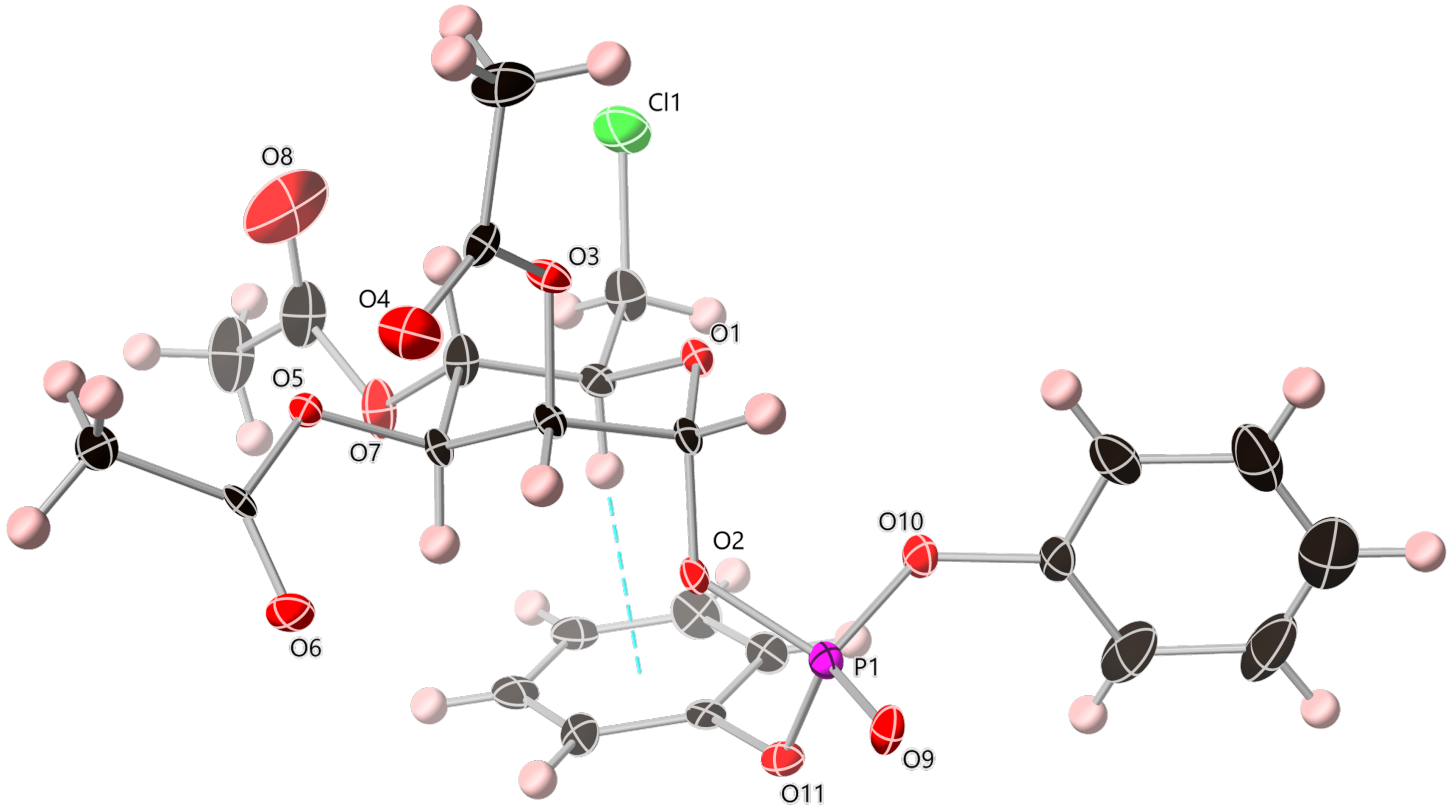
Structure of **16** with ADPs rendered at the 50% probability level. Acetyl group disorder is omitted for clarity. Atom colour scheme: carbon = black, oxygen = red, phosphorus = purple, chlorine = green, hydrogen = pink. C6–Cl is the *gg* rotamer (from the Cl6–C6–C5–O5 torsion angle).

Instead we started from **14** and completed a selective anomeric deacetylation on a gram-scale using ammonium acetate in DMF, to afford hemi-acetal **15** in good yield (80%) [11]. This was followed by phosphorylation of the anomeric position using diphenylphosphoryl chloride as the phosphorous electrophile, following deprotonation of **15** using *n*-BuLi at −78 °C. This afforded protected anomeric phosphate **16**, confirmed by ^1^H and ^13^C NMR data (H_1_ δ_H_ 6.01 ppm, ^3^*J*_H1–P_ = 6.7 Hz, ^3^*J*_H1–H2_ = 1.8 Hz; C_1_ δ_C_ 96.1 ppm, ^2^*J*_C1–P_ = 5.9 Hz). Following an initial purification of **16** using silica gel flash chromatography, the material was crystallised using a minimum volume of hot EtOH, enabling solid state analysis (Figure 2).

Crystals of **16** were analysed by single crystal X-ray diffraction and the data were solved in the monoclinic space group *P*2_1_. Although the crystals suffered from intrinsic non-merohedral twinning through a non-crystallographic rotation, the structural model allowed unambiguous assignment of the solid state molecular structure and absolute configuration. As shown in Figure 2, the desired axial anomeric phosphate was clearly visible alongside an interesting *gg* rotameric form for the C6–chloro side chain substituent (pyranose side chain conformation has recently been shown to be an important factor contributing to anomeric reactivity of canonical pyranoses [12]). One phenyl ring of the phosphate ester group occupied a position folded underneath the pyranose ring and engaged in an intramolecular C–H···π interaction with the axial C5 proton (Figure 2, dotted line). The C···π (mean plane) distance of 3.60 Å corresponded to an H···π distance of ca. 2.6 Å, consistent with the expected values for similar interactions among aryl-substituted monosaccharides [13–14]. The remaining weak interactions in the extended structure largely consist of diffuse intermolecular C–H···O contacts involving the phosphate and acetyl oxygen atoms.

Deprotection of **16** was completed in two steps, first using hydrogenolysis with Adam’s catalyst (PtO_2_), followed by removal of the acetate protecting groups with Et_3_N/H_2_O/MeOH, and furnished the target glycosyl 1-phosphate **17** as the sodium triethylamine salt. Overall, the synthesis of **17** was developed in 4 steps and 46% overall yield from **14**. With the required C6-modified glycosyl 1-phosphates in hand, we next evaluated them as substrates for enzymatic sugar nucleotide synthesis.

### Enzymatic synthesis of C6-modified sugar nucleotides

**Scheme 2:**
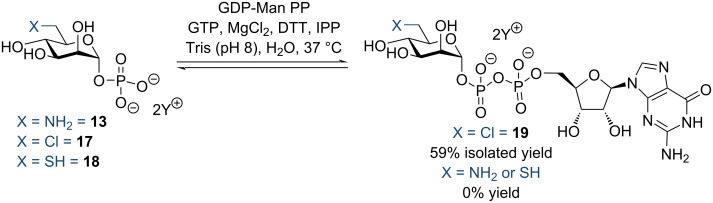
Evaluation of enzymatic GDP-Man synthesis using C6-modified mannose 1-phosphates **13**, **17**, and **18**; Y^+^ = appropriate counterion.

To evaluate pyrophosphorylative coupling using the C6-modified mannose 1-phosphates we selected the recombinant GDP-mannose pyrophosphorylase (GDP-Man PP) from *Salmonella enterica*, previously established to have a broad promiscuity for GDP-Man synthesis [4,6,15]. Glycosyl 1-phosphates **13**, **17**, and **18** were individually incubated with GDP-Man PP at 37 °C (Scheme 2) and the reactions monitored by TLC (iPrOH/NH_4_OH/H_2_O 6:3:1).

Monitoring of analytical scale reactions showed an indicative conversion for substrate **17** after 16 h; no conversion was observed after this time for **13** or **18** (see Figure S3 in Supporting Information File 1). Further analytical HPLC analysis for the formation of **19** from **17** after 16 h indicated a product had formed with a similar retention time to the control synthesis of **1**; this was tentatively assigned as **19**. Given this, we completed further enzymatic synthesis on a preparative scale to yield **19** in milligram quantities and in an isolated yield of 59%.

We previously demonstrated the GDP-Man-PP from *S. enterica* exhibited relaxed specificity towards C4 & C5 pyranose modification, although there is a fine balance to be struck between size and charge when modifying C6 [4]. Substrate **18** was known to form a disulfide in solution [10], presumably resulting in the glycosyl-1-phosphate being unable to access the enzyme active site; unfortunately, the addition of higher concentrations of reducing agent (DTT) and solid-supported PPh_3_ to access the reduced form for pyrophosphorylation had no positive effect. In the case of no observable reactivity for substrate **13**, likely protonation of the amine would result in unfavourable positive charge within the active site and the absence of vital H-bond contacts. X-ray crystallographic data of a related Man-PP from *T. maritima* show Asp260 & Asp109 in coordination with Mg^2+^ and two water molecules in close proximity to C6 [16].

### Evaluation of GDP 6-chloro-6-deoxy-ᴅ-mannose against GMD

**Figure 3:**
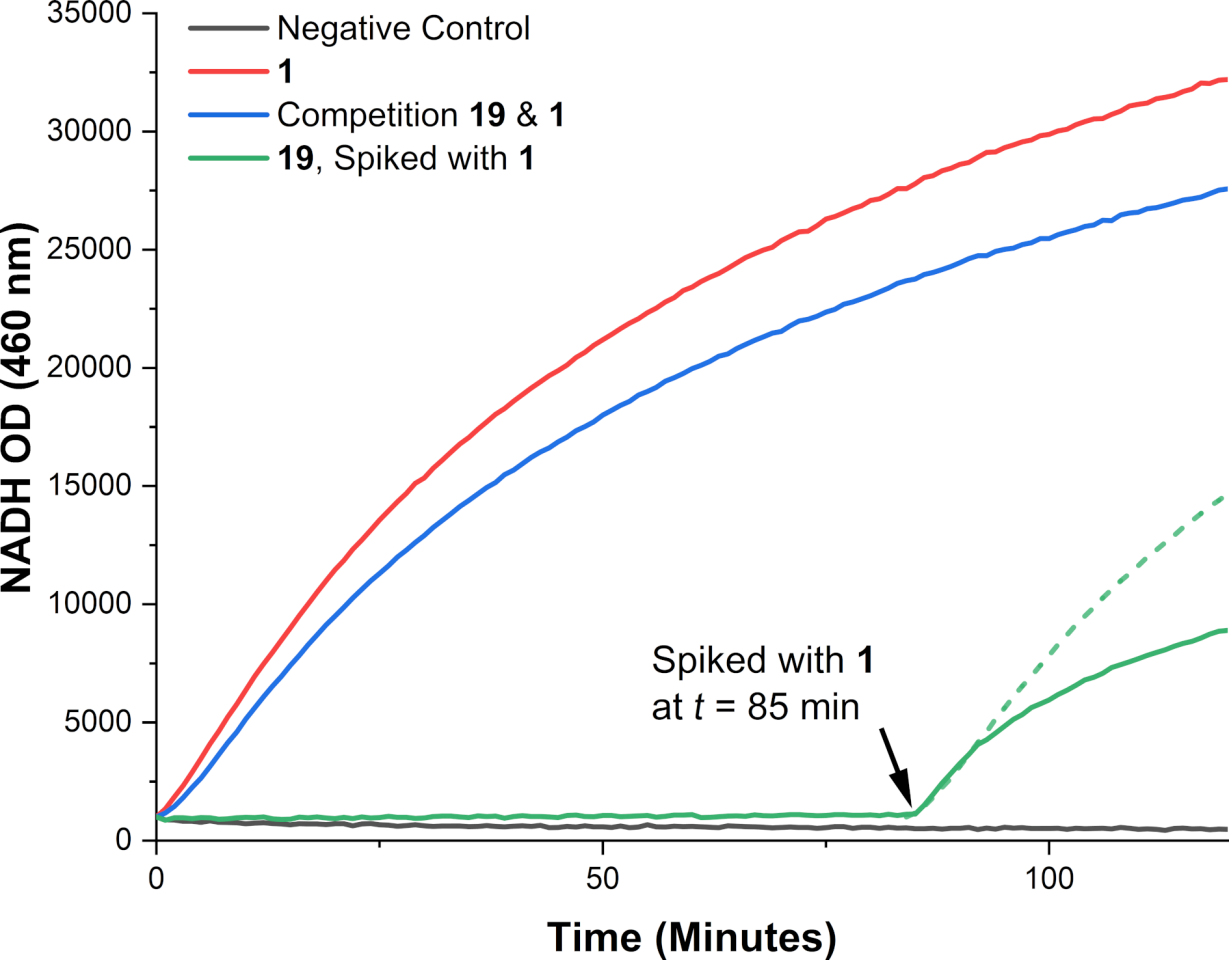
GMD function with probe **19** over 120 min (GMD (100 µg/mL), GDP sugars (50 µM), NAD^+^ (200 µM)). Dotted trace dictates expected fluorescence output following spiking with GDP-Man **1** if GMD activity fully restored (or probe **19** was not an inhibitor).

GMD from *P. aeruginosa* was co-incubated with GDP-mannose **1** and sugar nucleotide **19***,* and the production of NADH monitored by fluorescence (excitation 355 nm; emission 460 nm). Figure 3 illustrates a reduced rate of NADH production by GMD upon incubation with both probe **19** and the native substrate **1** (blue line). No significant increase in fluorescence was observed upon incubation of probe **19** and GMD (green line). However, a moderate level of NADH production was restored upon spiking the incubation with GDP-Man **1** after 85 minutes. The rate of fluorescence increase was reduced compared to both the positive control and incubation with **19** and **1** (Figure 3, blue line). This suggests that **19** may bind to GMD, but not as a substrate. Furthermore, we extended the GMD-**19** incubation time to overnight, followed by protein-MS analysis, but found no evidence of sugar nucleotide–protein conjugation; by contrast a positive control treating GMD with iodoacetamide showed multiple alkylation of the protein (see Figure S4 in Supporting Information Information File 1).

## Conclusion

We have developed a synthetic approach to a small series of C6-modified mannose 1-phosphates (6-amino, 6-chloro and 6-thio) and with these tools further explored the substrate promiscuity of the mannose pyrophosphorylase from *S.* enterica, observing that larger (than canonical OH) chlorine is accepted. Evaluation of GDP 6-chloro-6-deoxy-ᴅ-mannose suggests that the ligand can bind to GMD, but that targeting inhibitive *S*-alkylation of an sp^3^-hybridised C6 electrophilic probe is ineffective here.

## Supporting Information

File 1 Detailed experimental protocols and characterisation data; spectral NMR data (^1^H, ^13^C and ^31^P NMR for compounds **10**–**17** and **19**).
