# Supplementary material for: Synthesis of C6-modified mannose 1-phosphates and evaluation of derived sugar nucleotides against GDP-mannose dehydrogenase
Source: Beilstein J Org Chem. 2022 Sep 30;18:1379–84. doi: 10.3762/bjoc.18.142 (PMC9531554; doi:10.3762/bjoc.18.142)

BEILSTEIN JOURNAL OF ORGANIC CHEMISTRY

Supporting Information

for

Synthesis of C6-modified mannose 1-phosphates and evaluation of derived sugar nucleotides against GDP-mannose dehydrogenase

Sanaz Ahmadipour, Alice J. C. Wahart, Jonathan P. Dolan, Laura Beswick, Chris S. Hawes, Robert A. Field and Gavin J. Miller

Beilstein J. Org. Chem. **2022,** *18,* 1379–1384. doi:10.3762/bjoc.18.142

Detailed experimental protocols and characterisation data; spectral NMR data (^{1}H, ^{13}C and ^{31}P NMR for compounds 10–17 and 19)

License and Terms: This is a supporting information file under the terms of the Creative Commons Attribution License (https://creativecommons.org/licenses/by/4.0). Please note that the reuse, redistribution and reproduction in particular requires that the author(s) and source are credited and that individual graphics may be subject to special legal provisions.
The license is subject to the *Beilstein Journal of Organic Chemistry* terms and conditions: (https://www.beilstein-journals.org/bjoc/terms)

Table of contents

S1. General experimental

S2. Experimental procedures for compounds **10**–**17** and **19**

S3. Enzymatic synthesis of sugar nucleotides

S4. Evaluation of sugar nucleotide probes with GMD

S5. X-ray data

S6. References

S7. Spectral data: ^{1}H, ^{13}C and ^{31}P NMR for compounds **10**–**17** and **19**

S1. General experimental

All reagents and solvents which were available commercially were purchased from Acros, Alfa Aesar, Fisher Scientific, Sigma Aldrich or TCI. All reactions in non-aqueous solvents were conducted using oven-dried glassware with a magnetic stirring device under an inert atmosphere of nitrogen passed through a drying column using a vacuum manifold. Solvents were purified by passing through activated alumina columns and used directly from a Pure Solv-MD solvent purification system and were transferred under nitrogen unless otherwise stated. Reactions were followed by thin layer chromatography (TLC) using Merck silica gel 60 F_{254} analytical plates (aluminium support) and were developed using short wave UV radiation (245 nm) and/or 10% sulfuric acid in methanol/Δ. Purification *via* flash column chromatography was conducted manually using Sigma Aldrich silica gel 60 (0.040–0.063 mm) under a positive pressure of compressed air or *via* automation using a Büchi Reveleris X2 or a Büchi Pure C-815 Flash with pre-packed silica cartridges. Purification *via* strong ion exchange (SAX) chromatography was conducted using a Thermo Scientific™ HyperSep™ SAX 500 mg cartridge (column volume = 5 mL) with deionized water followed by aqueous NH_4HCO_3 (1.0 M). Purification *via* reversed phase separation was conducted using a Thermo Scientific™ HyperSep™ C18 cartridge (column volume = 5 mL) with deionized water followed by EtOAc and MeCN. Optical activities were recorded on an automatic Rudolph Autopol I or Bellingham and Stanley ADP430 polarimeter (concentration in g/100mL). ^{1}H NMR spectra were recorded at 400 MHz, ^{13}C NMR spectra at 100 MHz, and ^{31}P NMR spectra at 161 MHz respectively using Bruker Magnet system 400'54 Ascend. ^{1}H NMR resonances were assigned with the aid of

gDQCOSY. ^{13}C NMR resonances were assigned with the aid of gHSQCAD. Coupling constants are reported in hertz. Chemical shifts (δ, in ppm) are standardized against the deuterated solvent peak. NMR data were analyzed using Mestrenova. ^{1}H NMR splitting patterns were assigned as follows: br. s (broad singlet), s (singlet), d (doublet), dd (doublet of doublets), ddd (doublet of doublet of doublets), app. t (apparent triplet), t (triplet), quartet (q) or m (multiplet and/or multiple resonances). HRMS (ESI) were obtained on Agilent 6530 Q-TOF, LQT Orbitrap XL1 or Waters (Xevo, G2-XS TOF or G2-S ASAP) Micromass LCT spectrometers using a methanol mobile phase in positive/negative ionization modes, as appropriate.

S2. Experimental procedures for compounds 10-17 and 19

Synthesis of 6-amino-6-deoxy-α-D-mannose 1-phosphate 13

2,3,4-Tri-*O*-benzyl-6-bromo-6-deoxy-1-thio-α-D-mannopyranoside

To a solution of phenyl 2,3,4-tri-*O*-benzyl-1-thio-α-D-mannopyranoside[1] (**10**, 500 mg, 0.92 mmol, 1.0 equiv) in DCM (9 mL) at 0 °C was added successively Ph_3P (410 mg, 1.56 mmol, 1.7 equiv) and CBr_4 (520 mg, 1.56 mmol, 1.7 equiv), before warming to rt. After stirring for 16 h, the reaction mixture was poured onto H_2O (30 mL) and diluted with DCM (30 mL). The organic layer was washed with H_2O (2 × 30 mL), brine (30 mL), dried over $MgSO_4$, filtered, and concentrated *in vacuo*. Purification by silica gel column chromatography, eluting with pet. ether/EtOAc 6:1 afforded the title compound as a yellow oil (420 mg, 0.69 mmol, 75 %). $\mathbf{R_f}$ (Pet. Ether:EtOAc, 3:1) = 0.90; $[\alpha]^{26}_{D}$ +51.4 (*c* = 0.70 M, $CHCl_3$); **^{1}H NMR (400 MHz, $CDCl_3$)** δ 7.48-7.45 (m, 2H, Ar-*H*), 7.40-7.27 (m, 18H, Ar-*H*), 5.59 (d, $^{3}J_{H1-H2}$ = 1.5 Hz, 1H, H-1), 5.01 (d, $^{2}J_{CH-CH}$ = 10.9 Hz, 1H, CH_2Ph), 4.76-4.60 (m, 5H, CH_2Ph), 4.31-4.25 (m, 1H, H-5), 4.06-4.00 (m, 2H, H-2, H-4), 3.87 (dd, $^{3}J_{H3-H4}$ = 9.3 Hz, $^{3}J_{H3-H2}$ = 3.0 Hz, 1H, H-3), 3.69-3.66 (m, 2H, H-6a, H-6b); **^{13}C NMR (100 MHz, $CDCl_3$)** δ 138.3 (Ar-*C*), 138.1 (Ar-*C*), 137.9 (Ar-*C*), 134.2 (Ar- *C*), 131.8 (Ar-*C*), 129.2 (Ar-*C*), 128.6 (2C, Ar-*C*), 128.5 (Ar-*C*), 128.2 (Ar-*C*), 128.0 (3C, Ar-*C*), 127.9 (Ar-*C*), 127.7 (Ar-*C*), 86.0 (C-1), 80.1 (C-3), 76.9 (C-4), 76.3 (C-2), 75.6 (*C*H_2Ph), 72.3 (C-5), 72.2 (*C*H_2Ph), 72.1 (*C*H_2Ph), 33.4 (C-6); **HRMS *m/z*** (ESI^+) found: $(M+Na)^+$ 628.1184, $C_{33}H_{33}BrO_4S$ requires 628.1180.

6-Azido-2,3,4-tri-*O*-benzyl-6-deoxy-1-thio-α-D-mannopyranoside 11

To a solution of 2,3,4-tri-*O*-benzyl-6-bromo-6-deoxy-1-thio-α-D-mannopyranoside (360 mg, 0.59 mmol, 1.0 equiv) in DMF (4 mL) was added NaN_3 (77 mg, 1.18 mmol, 2.0 equiv). The

reaction mixture was heated to 75 °C and stirred for 18 h, before being cooled to rt, poured onto H_2O (15 mL), and extracted with EtOAc (30 mL). The organic layer was washed with saturated aqueous $Na_2S_2O_3$ solution (20 mL), H_2O (20 mL), brine (20 mL), dried over $MgSO_4$, filtered, and concentrated *in vacuo*. Purification by silica gel column chromatography, eluting with toluene/EtOAc (12:1, 9:1, 6:1) afforded **11** as a yellow oil (214 mg, 0.38 mmol, 64 %). $\mathbf{R_f}$ (Tol:EtOAc, 6:1) = 0.74; $[\alpha]^{26}_{D}$ +35.5 (*c* = 0.45 M, $CHCl_3$); **^{1}H NMR (400 MHz, $CDCl_3$)** δ 7.46-7.28 (m, 20H, Ar-*H*), 5.58 (d, $^3J_{H1-H2}$ = 1.6 Hz, 1H, H-1), 5.00 (d, $^2J_{CH-CH}$ = 11.0 Hz, 1H, C*H*$_2$Ph), 4.77-4.68 (m, 2H, C*H*$_2$Ph), 4.69-4.62 (m, 3H, C*H*$_2$Ph), 4.29-4.23 (1H, m, H-5), 4.03 (dd, $^3J_{H2-H3}$ = 2.9 Hz, $^3J_{H2-H1}$ = 1.6 Hz, 1H, H-2), 3.99 (app. t, $^3J_{H4-H3/H5}$ = 9.4 Hz, 1H, H-4), 3.88 (dd, $^3J_{H3-H4}$ = 9.4 Hz, $^3J_{H3-H2}$ = 2.9 Hz, 1H, H-3), 3.48 (m, 2H, H-6a, H-6b); **^{13}C NMR (100 MHz, $CDCl_3$)** δ 138.3 (Ar-*C*), 138.1 (Ar-*C*), 137.9 (Ar-*C*), 134.1 (Ar- *C*), 131.6 (Ar-*C*), 129.3 (Ar-*C*), 128.6 (2C, Ar-*C*), 128.2 (Ar-*C*), 128.1 (Ar-*C*), 128.0 (2C, Ar-*C*), 127.9 (Ar-*C*), 127.7 (Ar-*C*), 85.8 (C-1), 80.1 (C-3), 76.3 (C-2), 75.6 (*C*H$_2$Ph), 75.5 (C-4), 72.7 (C-5), 72.2 (2C, *C*H$_2$Ph), 51.6 (C-6); **HRMS *m/z*** (ESI+) found: $(M+Na)^+$ 590.2113, $C_{33}H_{33}N_3O_4S$ requires 590.2090.

Dibenzyl 6-azido-2,3,4-tri-*O*-benzyl-6-deoxy-α-D-mannopyranosyl phosphate 12

Thioglycoside **11** (196 mg, 0.26 mmol, 1.0 equiv) was dissolved in DCM (2.6 mL) and stirred with powdered 4 Å MS for 1 h. DBP (108 mg, 0.39 mmol, 1.5 equiv) was added and the reaction mixture stirred for a further 30 min before being cooled to −30 °C. NIS (88 mg, 0.39 mmol, 1.5 equiv) and AgOTf (20 mg, 78 µmol, 0.3 equiv) were added successively and the reaction mixture was stirred until TLC analysis indicated the reaction was complete (45 min). The reaction was quenched with Et_3N, filtered over Celite™ and diluted with DCM (20 mL). The organic layer was washed with saturated aqueous $Na_2S_2O_3$ solution (20 mL), saturated aqueous $NaHCO_3$ solution (20 mL), H_2O (20 mL), brine (20 mL), dried over $MgSO_4$, filtered, and concentrated *in vacuo.* Purification by silica gel column chromatography, eluting with pet. ether/EtOAc (5:1, 3:1, 2:1) afforded **12** as a yellow oil (124 mg, 0.16 mmol, 65 %). $\mathbf{R_f}$ (Tol:EtOAc, 6:1) = 0.44; $[\alpha]^{26}_{D}$ +53.6 (*c* = 0.2 M, $CHCl_3$); **^{1}H NMR (400 MHz, $CDCl_3$)** δ 7.34-7.28 (m, 23H, Ar-*H*), 7.26-7.24 (m, 2H, Ar-*H*), 5.70 (dd, $^3J_{H1-P}$ = 6.1 Hz, $^3J_{H1-H2}$ = 1.9 Hz, 1H, H-1), 5.08-4.89 (m, 5H, C*H*$_2$Ph), 4.63 (s, 2H, C*H*$_2$Ph), 4.56 (d, $^2J_{CH-CH}$ = 11.0 Hz, 1H, C*H*$_2$Ph), 4.48 (s, 2H, C*H*$_2$Ph), 3.95 (app. t, $^3J_{H4-H3/5}$ = 9.5 Hz, 1H, H-4), 3.83 (ddd, $^3J_{H5-H4}$ = 9.5 Hz, $^3J_{H5-H6a}$ = 4.5 Hz, $^3J_{H5-H6b}$ = 2.7 Hz, 1H, H-5), 3.78 (dd, $^3J_{H3-H4}$ = 9.5 Hz, $^3J_{H3-H2}$ = 3.0 Hz, 1H, H-3), 3.70-3.68 (m, 1H, H-2), 3.35-3.25 (m, 2H, H-6a, H-6b); **^{13}C{^{31}P} NMR (100 MHz, $CDCl_3$)** δ 138.1 (Ar-*C*), 138.0 (Ar-*C*),

137.7 (Ar-*C*), 135.6 (2C), 135.5 (2C, Ar-*C*), 128.7 (3C, Ar-*C*), 128.4 (3C, Ar-*C*), 128.1 (Ar-*C*), 128.0 (Ar- *C*), 127.9 (2C, Ar-*C*), 127.8 (2C, Ar-*C*), 127.7 (Ar-*C*), 96.0 (C-1), 78.6 (C-3), 75.3 (CH_2Ph), 74.3 (C-4), 74.2 (C-2), 73.3 (C-5), 72.8 (CH_2Ph), 72.1 (CH_2Ph), 69.6 (CH_2Ph), 69.5 (CH_2Ph), 51.0 (C-6); **^{31}P NMR (161 MHz, $CDCl_3$)** δ –2.80 (d, $^3J_{H1-P}$ = 6.1 Hz, 1P); **HRMS *m/z*** (ESI^+) found $(M+Na)^+$ 758.2647 $C_{41}H_{42}N_3O_8P$ requires 758.2608.

6-Amino-6-deoxy-α-D-mannopyranosyl phosphate (disodium salt) 13

A suspension of **12** (48 mg, 65 µmol, 1.0 equiv), Pd/C (10% loading, 11 mg, 11 µmol, 0.03 equiv per benzyl) and $Pd(OH)_2$/C (20% loading, 8 mg, 11 µmol, 0.03 equiv per benzyl) in EtOH/THF 2:1 (0.9/0.4 mL) and 0.1 M HCl (0.76 mL, 76 µmol, 1.18 equiv) were stirred vigorously under an atmosphere of H_2 for 24 h. The reaction mixture was filtered over Celite™ and washed with MeOH/water 2:1 then 1:1 (20 mL total) then passed through Dowex® 50W-X8 resin (Na^+ form) before being concentrated under reduced pressure. The resultant residue was re-suspended in D_2O and lyophilized to afford **13** as a white solid (15 mg, 58 µmol, 90 %). **R_f** (MeCN:H_2O (3:1 plus 3 drops AcOH) = 0.07; $[\alpha]^{26}_D$ +24.0 (*c* = 0.46 M, H_2O); **^{1}H NMR (400 MHz, D_2O)** δ 5.42 (d, $^3J_{H1-P}$ = 5.6 Hz, 1H, H-1), 3.99 (brs, 2H, H-2, H-3), 3.89 (app. d, ^3J = 8.3 Hz, 1H, H-5), 3.58 (app. t, $^3J_{H4-H3/H5}$ = 9.5 Hz, 1H, H-4), 3.46 (d, $^2J_{H6a-H6b}$ = 12.9 Hz, 1H, H-6a), 3.19-3.06 (m, 1H, H-6b); **^{13}C{^{31}P} NMR (100 MHz, D_2O)** δ 95.8 (C-1), 70.3 (C-2 or C-3), 70.2 (C-2 or C-3), 69.2 (C-4), 68.1 (C-5), 40.5 (C-6); **^{31}P NMR (161 MHz, D_2O)** δ –2.00 (d, J_{H1-P} = 5.6 Hz, 1P); **HRMS *m/z*** (ESI^-) found: $(M-H)^-$ 258.0388 $C_6H_{13}NO_8P$ requires 258.0378.

Synthesis of 6-chloro-6-deoxy-α-D-mannose 1-phosphate 17

2,3,4-Tri-*O*-acetyl-6-chloro-6-deoxy-α/β-D-mannopyranose 15

NH_4OAc (1.01 g, 13.1 mmol, 4.0 equiv) was added to a solution of 1,2,3,4-tetra-*O*-acetyl-6-chloro-6-deoxy-β-D-mannopyranose[2] (**14**, 1.20 g, 3.28 mmol, 1.0 equiv) in DMF (3 mL). The mixture was stirred for 42 h at rt. When the reaction was complete, as indicated by TLC (lower R_f spot), the remaining NH_4OAc was filtered off and the filtrate concentrated to dryness *in vacuo*. To remove residual DMF, the crude material was suspended in LiCl solution for 18 h and then extracted with EtOAc (5 × 20 mL). The combined organic layers were washed again with LiCl solution (5 × 20 mL), dried over $MgSO_4$, filtered and concentrated under reduced pressure to afford **15** (960 mg, 2.66 mmol, 80%) which was used without further purification. **R_f** (Hexane:EtOAc, 1:1) = 0.55; **^{1}H NMR (400 MHz, DMSO)** δ 7.40 (br.s 1H, OH), 5.20 (dd, $^3J_{H3-}$

$_{H4}$ = 10.1 Hz, $^3J_{H3-H2}$ = 3.3 Hz, 1H, H-3), 5.11 (t, $^3J_{H4-H3/5}$ = 9.8 Hz, 1H, H-4), 5.08 (m, 1H, H-1), 5.01 (dd, $^3J_{H2-H3}$ = 3.3 Hz, $^3J_{H2-H1}$ = 1.8 Hz, 1H, H-2), 4.16-4.11 (m, 1H, H-5), 3.77 (dd, $^2J_{H6a-H6b}$ = 12.1 Hz, $^3J_{H6a-H5}$ = 2.5 Hz, 1H, H-6a), 3.67 (dd, $^2J_{H6b-H6a}$ = 12.1 Hz, $^3J_{H6b-H5}$ = 5.6 Hz, 1H, H-6b), 2.09 (s, 3H, C*H*$_3$COO), 2.04 (s, 3H, C*H*$_3$COO), 1.93 (s, 3H, C*H*$_3$COO); **^{13}C NMR (100 MHz, DMSO)** δ 169.8 (*C*=O), 169.6 (*C*=O), 169.4 (*C*=O), 91.0 (C-1), 70.1 (C-2), 68.8 (C-5), 68.6 (C-3), 66.5 (C-4), 44.2 (C-6), 20.7 (*C*H$_3$), 20.5 (*C*H$_3$), 20.4 (*C*H$_3$)**; HRMS m/z** (NSI$^+$) found: (M+NH$_4$)$^+$ 342.0950, $C_{12}H_{21}ClNO_8$ requires 342.0950.

Diphenyl 2,3,4-tri-*O*-acetyl-6-chloro-6-deoxy-α-D-mannopyranosyl phosphate 16

n-BuLi (0.61 mL, 0.95 mmol, 1.59 M, 1.2 equiv) was added dropwise to a solution of **15** (261 mg, 0.80 mmol, 1.0 equiv) in THF (5 mL) at −78 °C. After stirring for 15 minutes, diphenyl phosphoryl chloride (0.20 ml, 0.95 mmol, 1.2 equiv) was added dropwise and the reaction mixture stirred for another 35 min at the same temperature. When TLC analysis indicated the complete consumption of the starting material (to a lower R_f spot), the reaction was gradually warmed to rt, quenched with saturated aqueous NH_4Cl solution (2 mL), and extracted with EtOAc (3 × 10 mL). The combined organic layers were washed with saturated aqueous NaCl solution (20 mL), dried over $MgSO_4$, filtered, and concentrated *in vacuo*. The residue was purified using silica gel *via* flash chromatography, eluting with DCM/Et_2O 1:0, 99:1, 98:2, 97:3 to afford **16** as a solid. This material was crystallized using a minimum amount of hot EtOH to further afford **16** as white crystals (260 mg, 0.47 mmol, 58%). **R_f** (DCM:Et_2O, 95:5) = 0.75; $[\alpha]^{25}_{D}$ +53.7 (*c* = 0.50, DCM); **^{1}H NMR (400 MHz, DMSO)** δ 7.47-7.43 (m, 4H, Ar), 7.31-7.27 (m, 6H, Ar), 6.01 (dd, $^3J_{H1-P}$ = 6.7 Hz, $^3J_{H1-H2}$ = 1.8 Hz, 1H, H-1), 5.27-5.25 (m, 1H, H-2), 5.23-5.17 (m, 2H, H-3, H-4), 4.11-4.07 (m, 1H, H-5), 3.72 (dd, 1H, $^2J_{H6a-H6b}$ = 12.5 Hz, $^3J_{H6a-H5}$ = 4.8 Hz, H-6a), 3.63 (dd, 1H, $^2J_{H6b-H6a}$ = 12.5 Hz, $^3J_{H6b-H5}$ = 2.6 Hz, H-6b), 2.10 (s, 3H, C*H*$_3$COO), 2.05 (s, 3H, C*H*$_3$COO), 1.96 (s, 3H, C*H*$_3$COO); **^{13}C NMR (100 MHz, DMSO)** δ 170.1 (*C*=O), 169.8 (*C*=O), 169.6 (*C*=O), 150.2 (C^{IV}), 150.1 (C^{IV}), 130.7 (C_{Ar}), 126.4 (C_{Ar}), 126.4 (C_{Ar}), 126.3 (C_{Ar}), 120.5 (C_{Ar}), 120.4 (C_{Ar}), 120.4 (C_{Ar}), 120.3 (C_{Ar}), 96.1 (d, $^3J_{H1-P}$ = 5.9 Hz, C-1), 71.8 (C-5), 68.3 (d, $^3J_{H1-P}$ = 10.7 Hz, C-2), 68.1 (C-3), 65.5 (C-4), 43.6 (C-6), 21.0 (*C*H$_3$), 20.9 (*C*H$_3$), 20.8 (*C*H$_3$); **^{31}P NMR (162 MHz, DMSO)** δ -14.34 (d, $^3J_{P-H1}$ = 6.6 Hz); **HRMS m/z** (NSI$^+$) found: (M+NH$_4$)$^+$ 574.1240, $C_{24}H_{26}ClO_9NH_4$ requires 574.1240.

6-Chloro-6-deoxy-α-D-mannopyranose 1-phosphate (sodium triethylammonium salt) 17

PtO_2 (16 mg, 0.07 mmol, 30 mol %) was added to a solution of **16** (130 mg, 0.23 mmol, 1.0 equiv) in EtOH (3 mL) and sodium bicarbonate (39 mg, 0.47 mmol, 2.0 equiv). The resulting mixture was stirred overnight at rt under an atmosphere of H_2 (1 atm, balloon). The reaction was monitored by TLC (hexane:EtOAc 1:1, R_f = 0.00 and MeCN:H_2O:NH_4OH 9:1:0.1, R_f = 0.55) and upon completion was filtered through Celite® and concentred *in vacuo*. The crude product was subjected to NMR to protecting group removal. **^{1}H NMR (400 MHz, MeOD)** δ 5.50 (dd, $^3J_{H1-P}$ = 7.6 Hz, $^3J_{H1-H2}$ = 1.3 Hz, 1H, H-1), 5.42-5.41 (m, 2H, H-3, H-4), 5.33 (app. s, 1H, H-2), 4.39 (dt, $^3J_{H5-H4}$ = 8.8 Hz, $^3J_{H5-H6}$ = 3.1 Hz, 1H, H-5), 3.81 (dd, $^2J_{H6a-H6b}$ = 12.3 Hz, $^3J_{H6a-H5}$ = 2.8 Hz, 1H, H-6a), 3.79 (dd, $^2J_{H6b-H6a}$ = 12.3 Hz, $^3J_{H6b-H5}$ = 3.2 Hz, 1H, H-6b), 2.14 (s, 3H, C*H₃*COO), 2.05 (s, 3H, C*H₃*COO), 1.95 (s, 3H, C*H₃*COO); **^{13}C NMR (100 MHz, MeOD)** δ 171.7 (*C*=O), 171.6 (*C*=O), 171.4 (*C*=O), 94.7 (d, $^3J_{H1-P}$ = 4.6 Hz, C-1), 71.4, 70.8, 68.0, 58.3, 44.6 (C-6), 20.6 (*C*H_3), 20.6 (*C*H_3), 18.4 (*C*H_3); **^{31}P NMR (162 MHz, MeOD)** δ -0.50 (d, $^3J_{P-H1}$ = 7.1 Hz). Et_3N (1 mL) was added to the above crude in MeOH (2 mL), and the solvent removed *in vacuo*. The residue was dissolved in Et_3N:H_2O:MeOH 1:3:7 (*v/v/v*, 5 mL) and stirred for 26 h at rt. TLC analysis (MeCN:H_2O:NH_4OH 9:1:0.1, R_f = 0.00) showed complete conversion of starting material and the mixture was concentrated *in vacuo*. The crude was dissolved in water (2 mL), stirred for 1 h at rt with ion exchange resin (Amberlite® IR120 Na^+ form), filtered and the filtrate freeze dried to afford crude **17** as a white powder. This material was purified using a RP-C18 column, eluting with H_2O (2CV), EtOAc (2CV) and MeCN (2CV). The product containing fractions were collected and freeze dried to afford **17** as a white powder (91 mg, 0.23 mmol, 99 %). $[\alpha]^{24.6}_D$ +20.7 (*c* = 0.45, H_2O); **^{1}H NMR (400 MHz, D_2O)** δ 5.28 (d, $^3J_{H1-P}$ = 7.3 Hz, 1H, H-1), 4.00 (dt, $^3J_{H5-H4}$ = 9.6 Hz, $^3J_{H5-H6}$ = 2.9 Hz, 1H, H-5), 3.91 (br.s, 1H, H-2), 3.88-3.85 (m, 3H, H-3, H-6a, H-6b), 3.76 (t, $^3J_{H4-H3/H5}$ = 9.5 Hz, 1H, H-4), 3.13 (q, 6H, $^3J_{CH2-CH3}$ = 6.5 Hz, C*H₂*-NEt₃), 1.21 (t, $^3J_{CH3-CH2}$ = 6.9 Hz, 9 H, C*H₃*-NEt₃); **^{13}C NMR (100 MHz, D_2O)** δ 95.3 (d, $^2J_{C-P}$ = 4.7 Hz, C-1), 71.5 (C-5), 70.8 (d, $^3J_{C-P}$ = 7.4 Hz, C-2), 68.8 (C-3), 67.1 (C-4), 46.6 (*C*H_2-NEt_3), 44.6 (C-6), 8.2 (*C*H_3-NEt_3); **^{13}C-GATED (101 MHz; D_2O):** δ 98.5 ($^1J_{C1-H1}$ = 170.0 Hz, C-1α); **^{31}P NMR (162 MHz, D_2O)** δ 0.79 (br.s). **HRMS m/z** (NSI^-) found $(M-H)^-$ 276.9887, $C_6H_{11}ClO_8P$ requires 276.9886.

S3 Enzymatic synthesis of sugar nucleotides

Expression and purification of GDP-mannose-pyrophosphorylase (GDP-Man-PP)

The transformant was grown according to the literature.[3] Briefly, 1 L of transformant in LB medium containing appropriate antibiotic (kanamycin, 25 µg/mL) was incubated at 37 °C with gentle shaking until an OD_{600} of about 0.6. Heterologous protein expression was induced by adding isopropyl β-D-1-thiogalactopyranoside (IPTG) at 0.5 mM final concentration, followed by incubation at 18 °C overnight at 180 rpm. Afterwards, cells were harvested by centrifugation (4000 × *g*, 4 °C, 20 min) and stored at −80 °C until use. Frozen cells were thawed in 50 mM Tris-HCl pH 8.0, 500 mM NaCl, 20 mM imidazole supplemented with DNase (10 µg/mL, Sigma) and proteinase inhibitor cocktail (Roche), then lysed by sonication in ice. After centrifugation (20,000 *x g*, 4 °C, 20 min) to remove the cell debris, the crude protein solution was purified at 4 °C using an ÄKTA pure FPLC system (GE Healthcare). The supernatant was passed through a HisTrapTM HP column (5 mL, GE healthcare), pre-equilibrated with buffer A (50 mM Tris-HCl pH 8.0, 500 mM NaCl, 20 mM imidazole). Unbound proteins were washed with five column volumes of buffer A, followed by elution with buffer B (50 mM Tris-HCl pH 8, 500 mM NaCl, 500 mM imidazole). GDP-man-PP comprising fractions were pooled together and concentrated to ≈7 mg/mL (concentration determined by Pierce™ BCA assay, ThermoFisher or Bradford assay, Sigma). Concentrated GDP-man-PP was then divided into aliquots and stored at −80 °C until required.

Evaluation of C6-modified glycosyl 1-phosphates

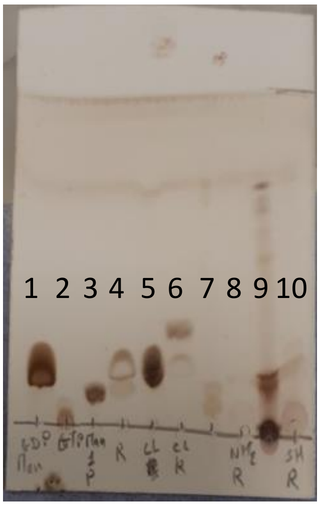

Figure S1: TLC of GDP-man-PP catalyzed guanylyltransfer of GMP from GTP to 6Cl-Man-1P **17**, 6SH-Man-1P **18**, and 6NH_2-Man-1P **13** after 16 h at 37 °C. No reaction is observed for 6-thio **18** or 6-amino **13**. Lane 1: GDP-Man (authentic); Lane 2: GTP; Lane 3: Man-1P; Lane 4: Man-1P guanylyltransfer reference reaction; Lane 5: 6Cl-Man-1P **17**; Lane 6: 6Cl-Man-1P guanylyltransfer reaction; Lane 7: 6NH_2-Man-1P **13**; Lane 8: 6NH_2-Man-1P guanylyltransfer reaction; Lane 9: 6SH-Man-1P **18**; Lane 10: 6SH-Man-1P guanylyltransfer reaction.

No reaction was observed for 6-thio Man-1P **18** or 6-amino Man-1P **13**. The 6-thio substrate was found to form a disulfide in solution; increasing the concentration of reducing agent within the reaction from 1 mM to 20 mM had no effect on reaction progression. The addition of solid supported PPh_3 to stabilize the reduced form or the addition of 20 mM DTT also had no effect on reaction progression.

Guanosine diphosphate-6-chloro-6-deoxy-α-D-mannose 19

The enzymatic synthesis of sugar-nucleotides by GDP-Man-PP was completed as follows: The buffer was Tris-HCl (pH 8.0, 40 mM) containing $MgCl_2$ (8 mM) and DTT (1 mM). The final concentrations were as follows: glycosyl 1-phosphate **17** (7.5 mg, 18.7 µmol, 1.0 equiv, 6.0 mM) and GTP (10.59 mg, 20.2 µmol, 1.68 equiv). The enzyme concentrations were as follows: GDP-Man-PP (0.6 mg/mL) and inorganic pyrophosphatase (iPPase, Sigma, 2.70 U/mL). The reaction was incubated with shaking at 37 °C until formation of an NDP-sugar was observed by TLC (IPA/NH_4OH/H_2O 6:3:1). MeOH (213 µL) was added and the mixture was

centrifuged (9300 rpm) for 2 min to remove insoluble protein, passed through a syringe filter (0.4 µM, PTFE) and purified by SAX chromatography ThermoFisher Dionex UltiMate 3000 HPLC system using a Poros HQ 50 SAX column (5 mL), flow rate (7.0 mL/min), 5 → 250 mM NH_4HCO_3 over 15 min with in-line UV detector to monitor at 265 nm, to afford **19** as a white solid (6.9 mg, 11.0 µmol, 59%).

^{1}H NMR (500 MHz, D_2O) δ 8.12 (s, 1H, H-8”), 5.93 (d, $^3J_{H1'\text{-}H2'}$ = 6.1 Hz, 1H, H-1’), 5.49 (dd, $^3J_{H1\text{-}P}$ = 7.6 Hz, $^3J_{H1\text{-}H2}$ = 1.2 Hz, 1H, H-1), 4.77 (s, hidden, H-2'), 4.51 (dd, $^3J_{H3'\text{-}H2'}$ = 5.1 Hz, $^3J_{H3'\text{-}H4'}$ = 3.5 Hz, 1H, H-3'), 4.35 (dd, $^3J_{H4'\text{-}H3'}$ = 3.1 Hz, $^3J_{H4'\text{-}H5'}$ = 1.8 Hz, 1H, H-4'), 4.21 (dd, J = 5.2 Hz, J = 3.8 Hz, 2H, H-5'), 4.08 – 4.04 (m, 2H, H-4, H-2), 3.94 (dd, $^3J_{H3\text{-}H4}$ = 9.9 Hz, $^3J_{H3\text{-}H2}$ = 3.4 Hz, 1H, H-3), 3.91-3.85 (m, 2H, H-5, H-6a), 3.84-3.80 (m, 1H, H-6b); **^{13}C NMR (125 MHz, D_2O)** δ 96.5 (C-1), 86.8 (C-1'), 83.7 (C-4'), 73.5 (C-2'), 72.1 (C-4), 70.3 (C-3'), 70.1 (C-2), 69.5 (C-3), 66.6 (C-5), 62.6 (C-5’), 44.2 (C-6); **^{31}P{^{1}H} NMR (200 MHz, D_2O)** δ −11.50 (d, $^2J_{C\text{-}P}$ = 24.0 Hz), −14.01 (d, $^2J_{C\text{-}P}$ = 25.1 Hz); **HRMS *m/z*** (NSI-) found $(M\text{-}H)^-$ 622.0335, $C_{16}H_{23}ClN_5O_{15}P_2$ requires 622.0360.

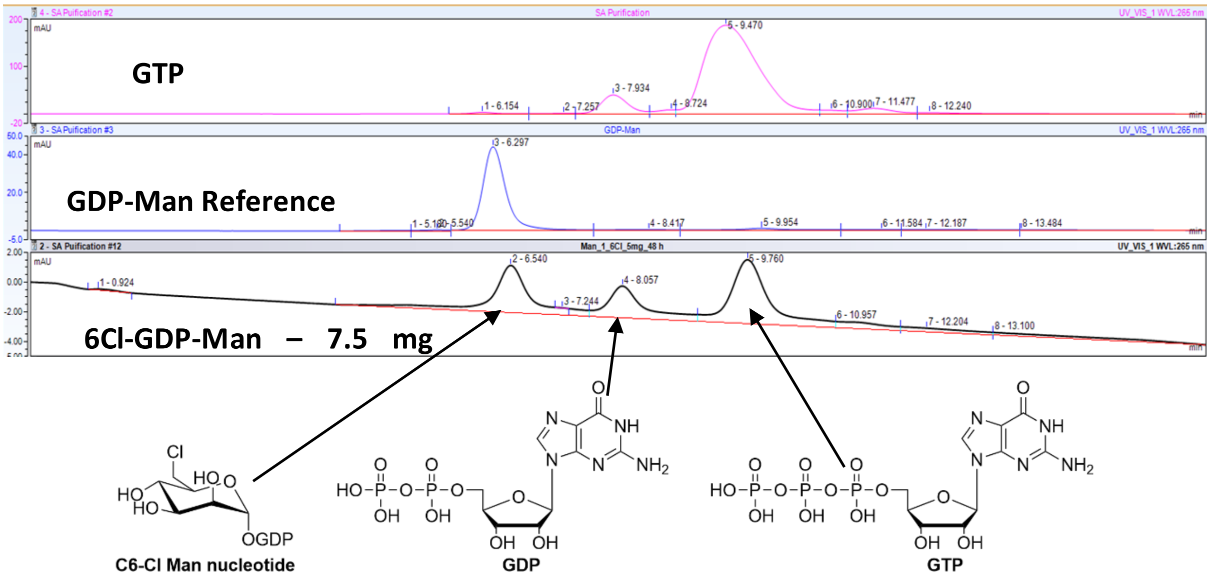

Figure S2: HPLC Purification of 6-Cl GDP-Man **19** after 16 hours using ThermoFisher Dionex UltiMate 3000 HPLC system using a Poros HQ 50 SAX column (5 mL), flow rate (7.0 mL/min), 5 → 250 mM NH_4HCO_3 over 15 min. HPLC after 16 hours reaction indicated presence of GTP (9.377 min), presence of GDP (7.800 min) and presence of the desired nucleotide **9** (6.124 min).

S4. Evaluation of sugar nucleotide probes with GMD

Expression and purification of GMD from *P. aeruginosa*

The recombinant plasmid (pET-3a) containing the *algD* gene encoding for GDP-mannose dehydrogenase (GMD) from *P. aeruginosa* was kindly donated by P. Tipton. The plasmid was transformed into *E. coli* soluBL21(DE3) chemically competent cells and the transformant grown according to the literature.[2,4] Briefly, 1 L of the transformant in LB medium containing the appropriate antibiotic (carbenicillin, 100 µg/mL) was incubated at 37 °C with gentle shaking in baffled flasks until an OD_{600} of 0.6–0.8 was reached. Heterologous protein expression was induced by adding isopropyl β-D-1-thiogalactopyranoside (IPTG) to a final concentration of 0.4 mM, followed by incubation at 37 °C for 4 hours at 180 rpm. Afterwards the cells were harvested by centrifugation (4000 *x g*, 4 °C, 20 min) and stored at −80 °C until use. Frozen cells were thawed in 20 mM HEPES (pH 7.5), 150 mM NaCl supplemented with DNase A (10 µg/mL, Sigma) and proteinase inhibitor cocktail (Roche), then lysed by sonication on ice. The supernatant was recovered by centrifugation (20,000 *x g*, 4 °C, 20 min) and nucleic acid precipitated through the addition of protamine sulfate (5 mg per gram wet cell pellet) and incubated on ice for 30 min. Precipitated nucleic acid removed by centrifugation (20,000 *x g*, 4 °C, 20 min), the crude protein solution was fractionated with ammonium sulfate, with GMD precipitating between 45 and 60% saturation. Protein pellets were redissolved in 20 mM HEPES (pH 7.5), 150 mM NaCl and purified using an ÄKTA pure FPLC system (GE Healthcare) by gel filtration chromatography using a Superdex S200 16/600 column (GE Healthcare). Proteins were eluted with 20 mM HEPES (pH 7.5) and 150 mM NaCl at the flow rate of 1 mL/min. GMD containing fractions were combined and concentrated to ≈4.5 mg/mL (concentration determined by Pierce™ BCA assay, ThermoFisher or Bradfords Assay, Sigma). Concentrated GMD was then divided into aliquots and stored at −80°C until required in 10% glycerol.

GMD inhibition assay

Assay protocol

The assay was performed in 96-well flat bottomed, non-binding, polystyrene microtiter plates (Grenier 655906). NAD^+ (200 µM), **19** (50 µM) and GMD (25 or 50 µg/mL) were prepared in 50 mM sodium phosphate (pH 7.4) containing 0.5 mM $MgCl_2$ and 1 mM DTT. A solution of

GDP-Man (final: 10 µM) was added to the plate and the fluorescence was measured at 25 °C for 65 minutes using a BMG labtech FLUOStar Omega microplate reader (excitation 355 nm; emission 460 nm). The limits of detection were analyzed by control samples as followed: positive control contained no inhibitor; negative control contained no inhibitor or GMD.

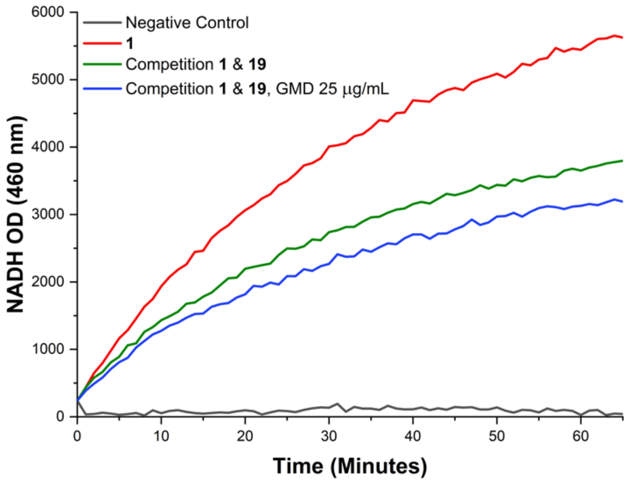

Figure S3: GMD function with probe **19** (50 µM) over 65 minutes. GMD (50 µg/mL, unless stated), GDP-Man **1** (50 µM), NAD$^+$ (200 µM). Negative control experiment was run with no GMD.

GMD alkylation by iodoacetamide

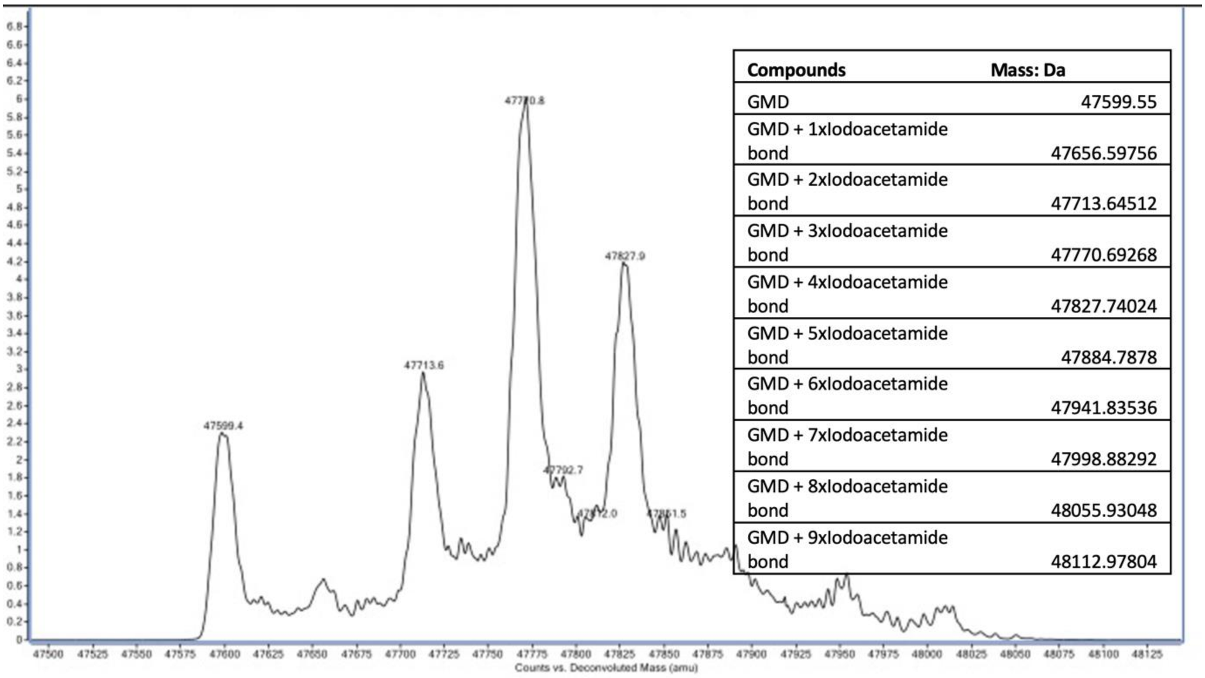

Compounds	Mass: Da
GMD	47599.55
GMD + 1xIodoacetamide bond	47656.59756
GMD + 2xIodoacetamide bond	47713.64512
GMD + 3xIodoacetamide bond	47770.69268
GMD + 4xIodoacetamide bond	47827.74024
GMD + 5xIodoacetamide bond	47884.7878
GMD + 6xIodoacetamide bond	47941.83536
GMD + 7xIodoacetamide bond	47998.88292
GMD + 8xIodoacetamide bond	48055.93048
GMD + 9xIodoacetamide bond	48112.97804

Figure S4: Deconvoluted protein LC–MS of GMD following overnight incubation with iodoacetamide (10 equiv) showing multiple surface-exposed alkylation sites.

S5. X-Ray crystallography data

Crystal and refinement parameters are given in Table S1. All data were collected on a Bruker D8 Quest ECO diffractometer using graphite-monochromated Mo Kα radiation (λ = 0.71073 Å) and a Photon II-C14 CPAD detector. Crystals were mounted on Mitegen micromounts in NVH immersion oil, and all collections were carried out at 150 K using an Oxford cryostream. Data collections were carried out using ϕ and ω scans, with collections and data reductions carried out in the Bruker APEX-3 suite of programs.[5] Multi-scan absorption corrections were applied for all datasets using SADABS unless otherwise stated.[6] The data were solved with the intrinsic phasing routine in SHELXT,[7] and all data were refined on F^2 with full-matrix least squares procedures in SHELXL,[8] operating within the OLEX-2 GUI.[9] All non-hydrogen atoms were refined with anisotropic displacement parameters. Carbon-bound hydrogen atoms were placed in riding positions and refined with isotropic displacement parameters equal to 1.2 or 1.5 times the isotropic equivalent of their carrier atom. Crystals of **16** exhibited unavoidable non-merohedral twinning related by a 180 degree rotation which could not be mechanically separated. The two domains were indexed and their contributions to each reflection were separated using TWINABS,[10] and the final refinement was performed on the HKLF5 file with a batch scale factor of 0.45. A global RIGU restraint and localised ISOR restraints were necessary to avoid non-positive definite ADPs in the final refinement caused by the substantial overlap of the two lattices and resulting impact on the data quality. CCDC 2165925

Table S1 Crystal data and structure refinement for 16

Identification code	**16**
Empirical formula	$C_{24}H_{26}ClO_{11}P$
Formula weight	556.87
Temperature/K	150.0
Crystal system	monoclinic
Space group	$P2_1$
a/Å	11.9261(4)
b/Å	8.1862(3)
c/Å	14.2882(5)

α/°	90
β/°	109.912(2)
γ/°	90
Volume/Å^3	1311.55(8)
Z	2
ρ_{calc}g/cm^3	1.410
μ/mm^{-1}	0.265
F(000)	580.0
Crystal size/mm^3	0.19 × 0.07 × 0.03
Radiation	MoKα (λ = 0.71073)
2Θ range for data collection/°	5.468 to 50.992
Index ranges	-14 ≤ h ≤ 14, -9 ≤ k ≤ 9, -17 ≤ l ≤ 17
Reflections collected	43101 [17377 with I>=2σ (I)]
Independent reflections	4867 [$R_{int(HKLF4)}$ = 0.1235, R_{sigma} = 0.1014]
Data/restraints/parameters	4867/329/364
Goodness-of-fit on F^2	1.216
Final R indexes [I>=2σ (I)]	R_1 = 0.0919, wR_2 = 0.1285
Final R indexes [all data]	R_1 = 0.1091, wR_2 = 0.1342
Largest diff. peak/hole / e Å^{-3}	0.69/-0.76
Flack parameter	-0.04(5)
CCDC Number	2165925

S6. References

1. Ahmadipour, S. *et al*. *Org. Lett.*, 2019, **21**, 4415–4419.
2. Beswick, L. *et al*. *Carbohydr. Res.,* 2020, **488**, 107896.
3. Verseck et al, *Glycobiology*, 1996, **6**, 591–597.
4. Tipton et al, *Biochemistry*, 2002, **41**, 9637–9645.
5. *Bruker APEX-3*, Bruker-AXS Inc., Madison, WI, 2016.
6. *SADABS*, Bruker-AXS Inc., Madison, WI, 2016.
7. G. M. Sheldrick, *Acta Crystallogr., Sect. A: Found. Adv.*, 2015, **71**, 3–8.
8. G. M. Sheldrick, *Acta Crystallogr., Sect. C: Struct. Chem.*, 2015, **71**, 3–8.
9. O. V. Dolomanov, L. J. Bourhis, R. J. Gildea, J. A. K. Howard and H. Puschmann, *J. Appl. Crystallogr.*, 2009, **42**, 339–341.
10. *TWINABS*, Bruker-AXS Inc., Madison, WI, 2016.

S7. Spectral Data: ^{1}H, ^{13}C and ^{31}P NMR for compounds 10-17 and 19

2,3,4-Tri-*O*-benzyl-6-bromo-6-deoxy-1-thio-α-D-mannopyranoside

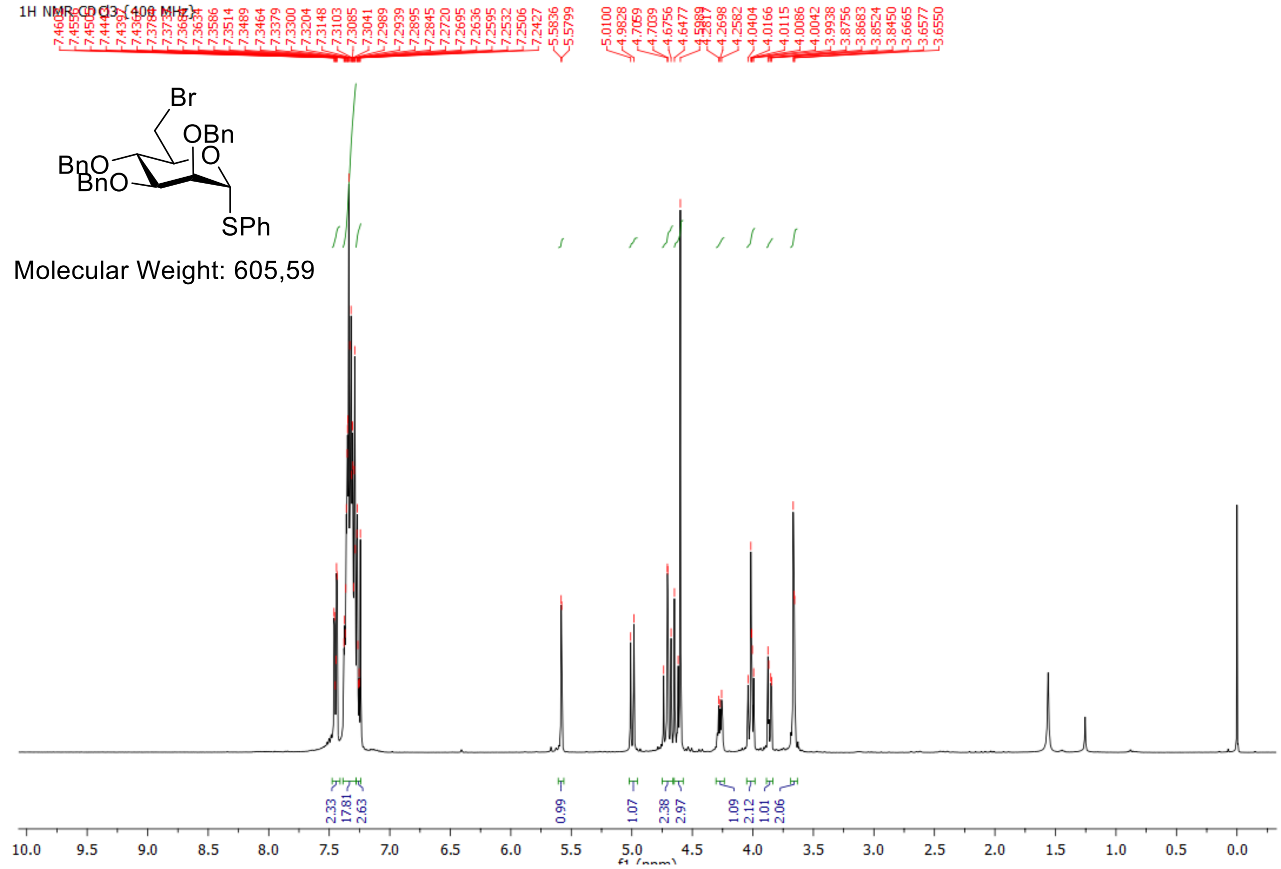

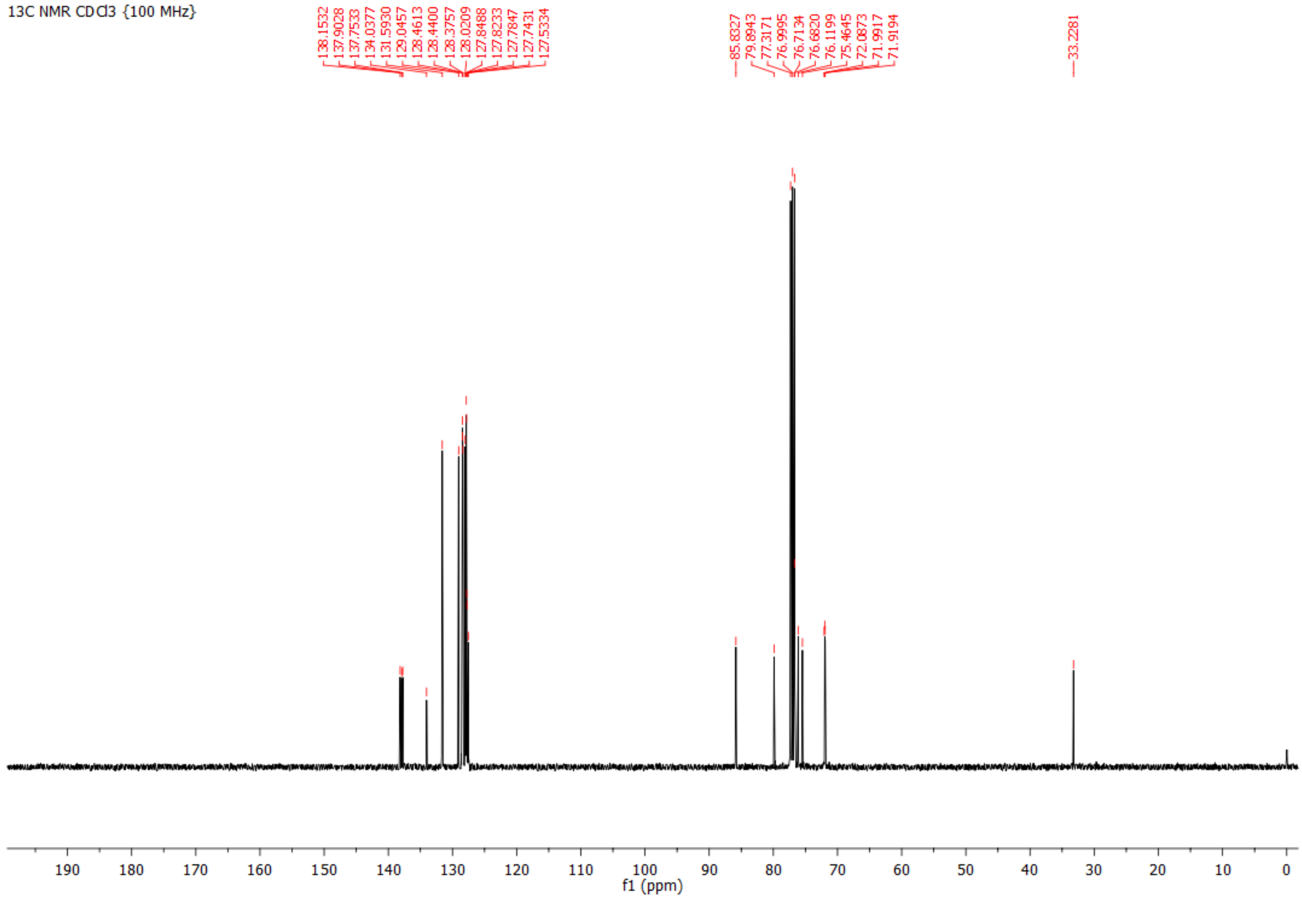

6-Azido-6-deoxy-2,3,4-tri-*O*-benzyl-1-thio-α-D-mannopyranoside 11

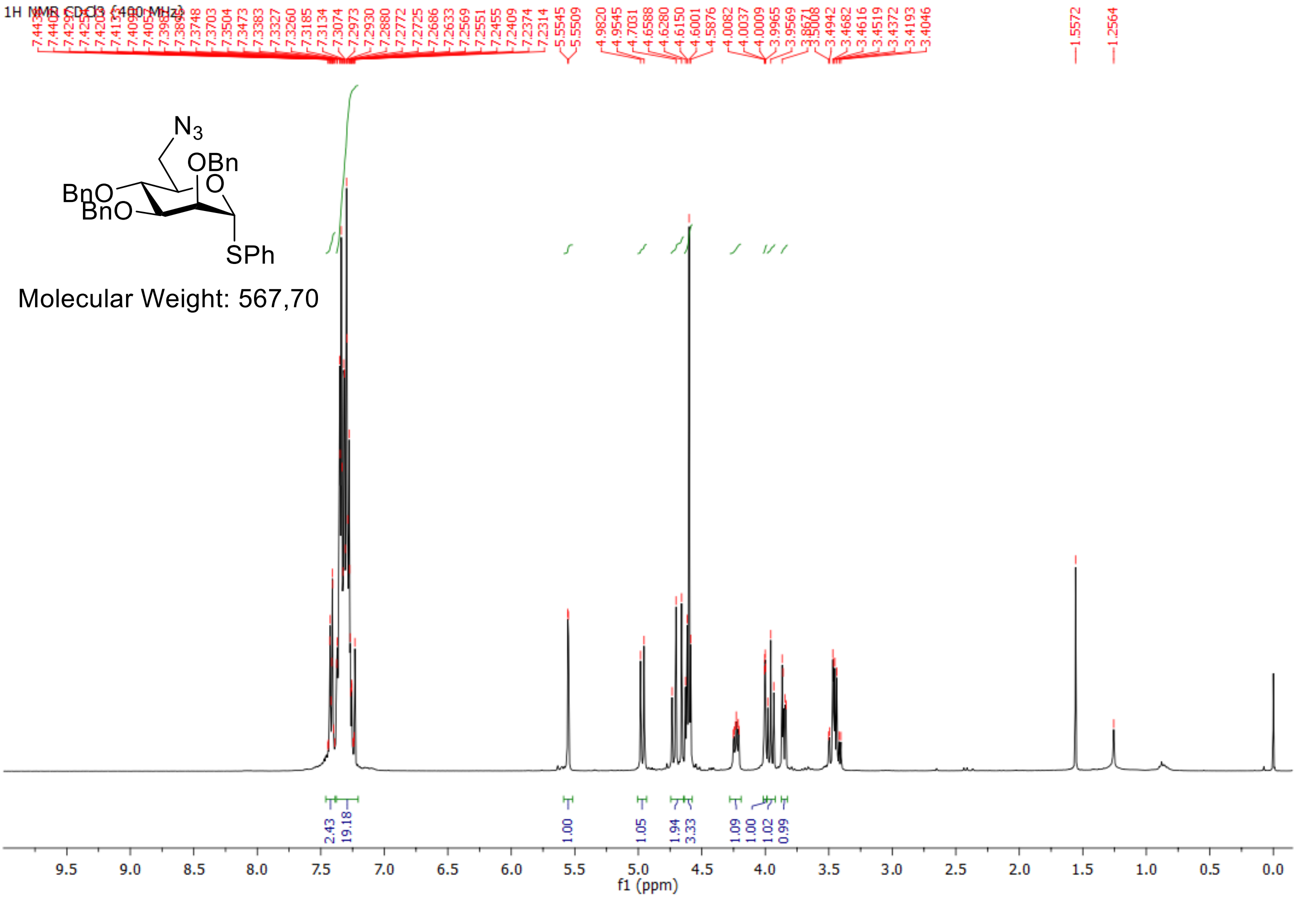

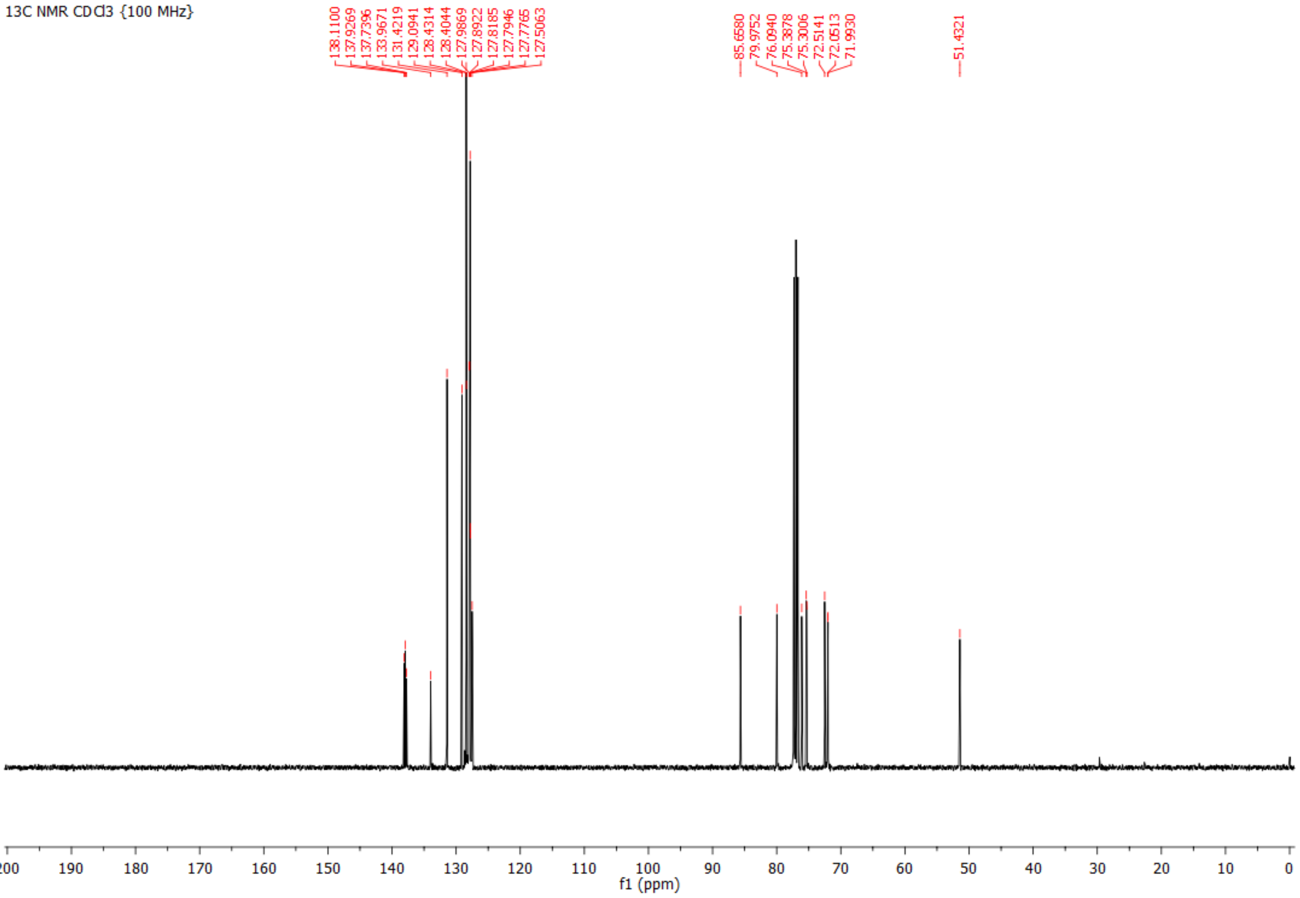

Dibenzyl 6-azido-6-deoxy-2,3,4-tri-*O*-benzyl-α-D-mannopyranosyl phosphate 12

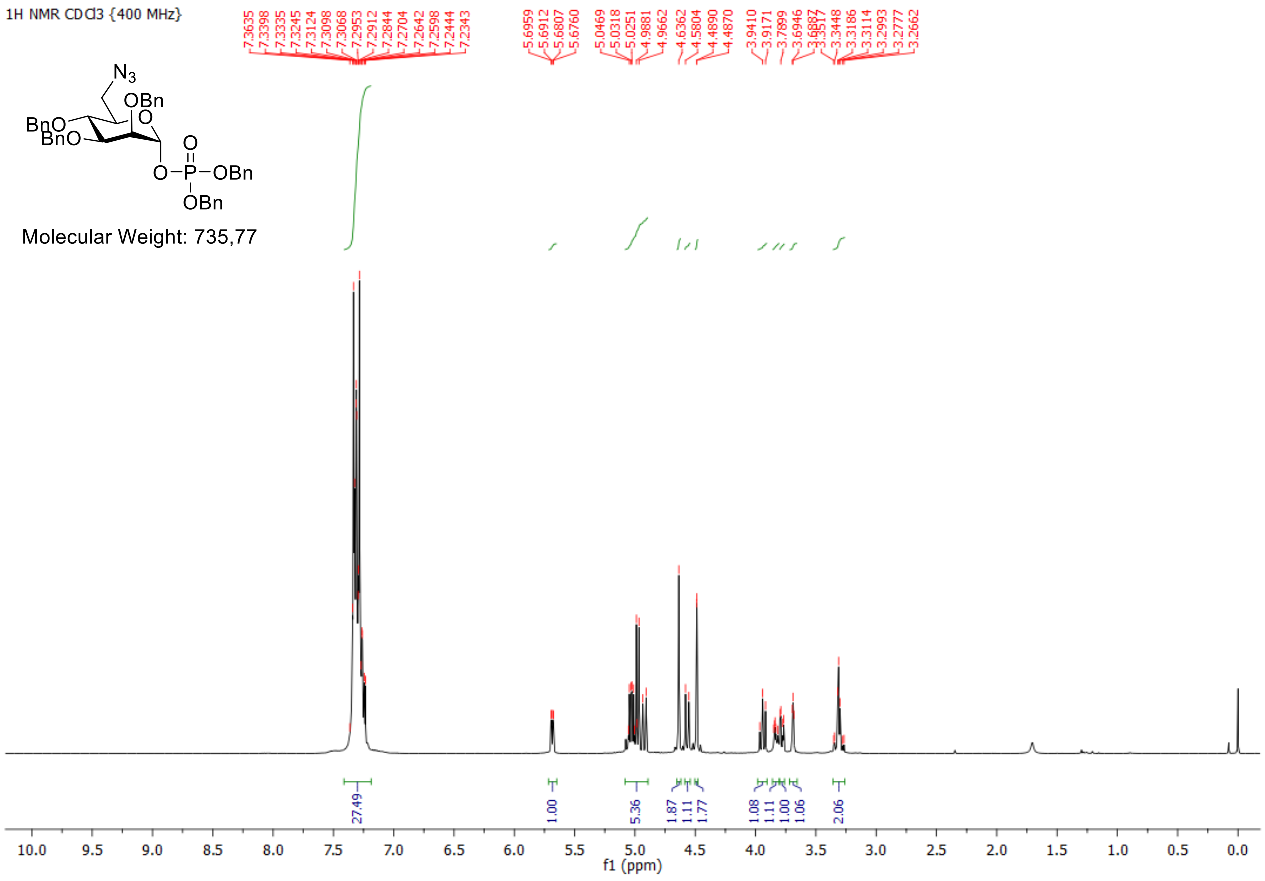

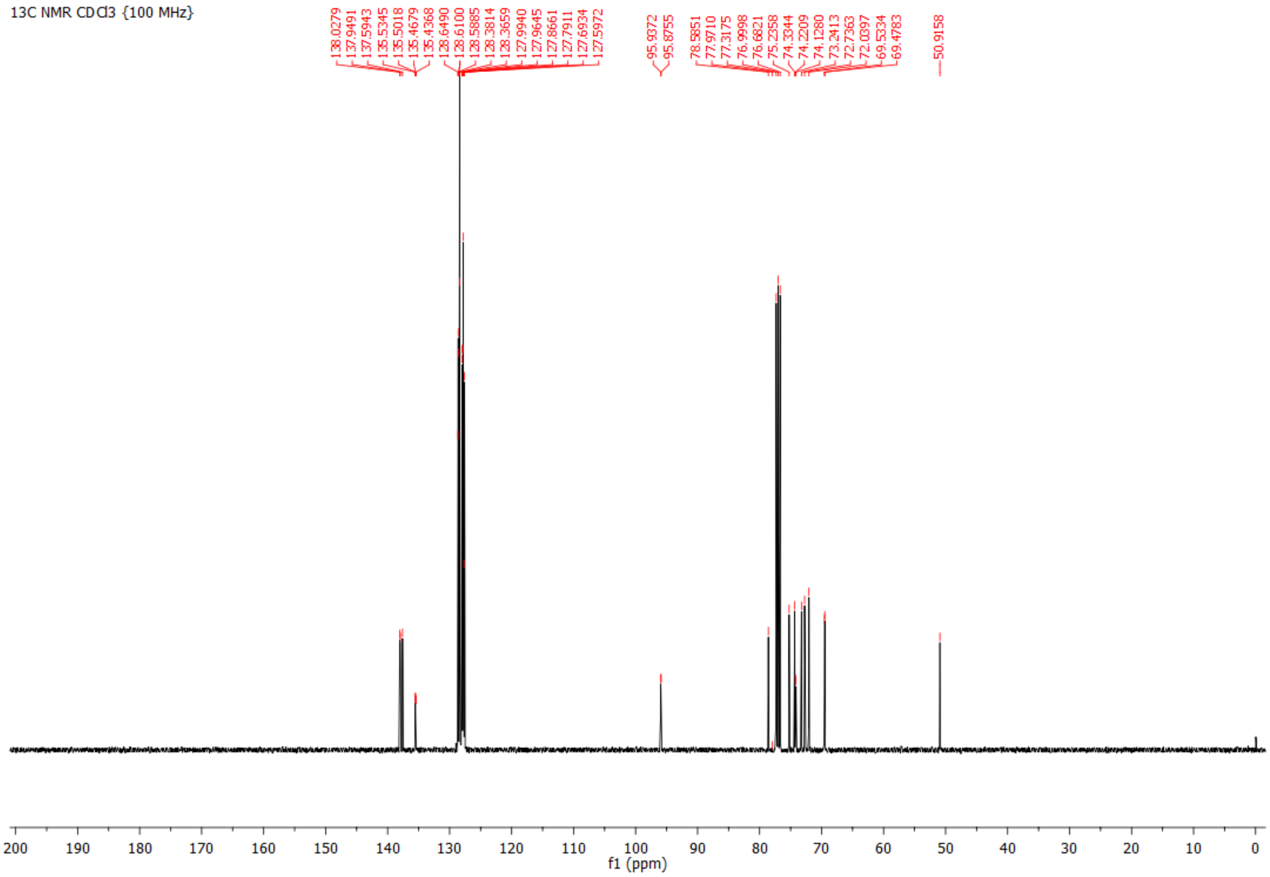

6-Amino-6-deoxy-α-D-mannopyranose 1-phosphate (disodium salt) 13

1H NMR D2O {400 MHz}

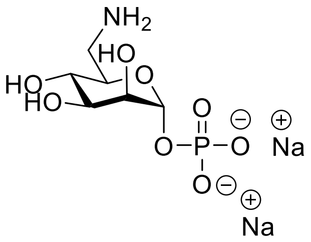

Molecular Weight: 303,11

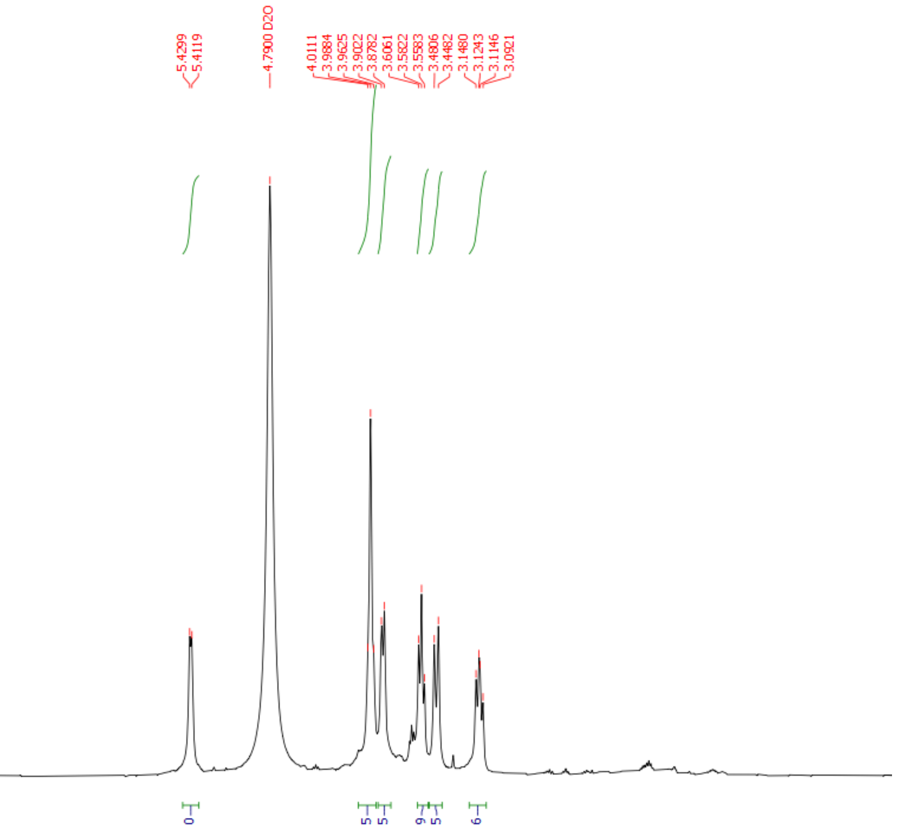

13C NMR D2O {100 MHz}

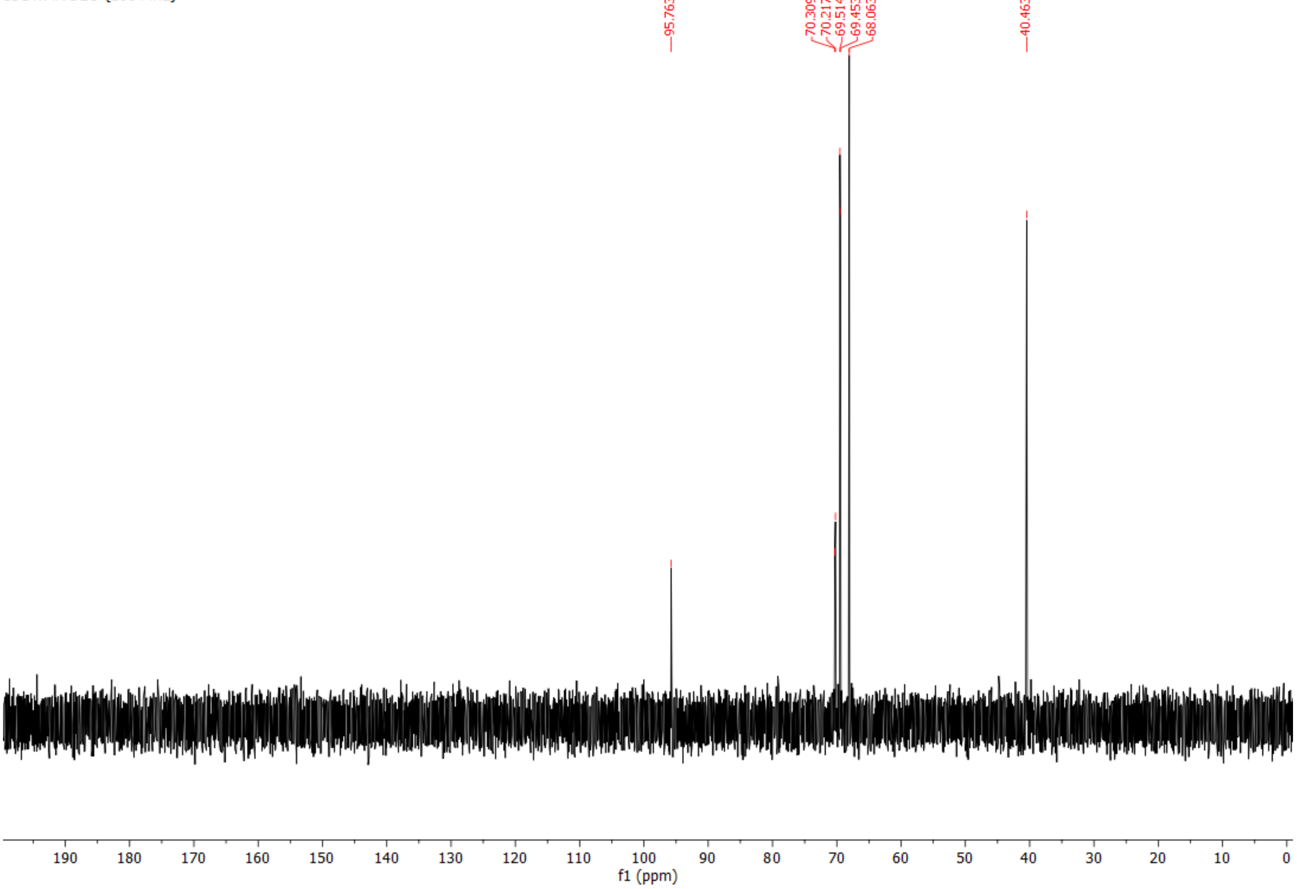

2,3,4-Tri-*O*-Acetyl 6-chloro-6-deoxy-D-mannopyranose 15

1H NMR DMSO {400 MHz}

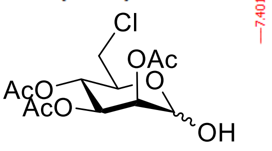

Molecular Weight: 324,71

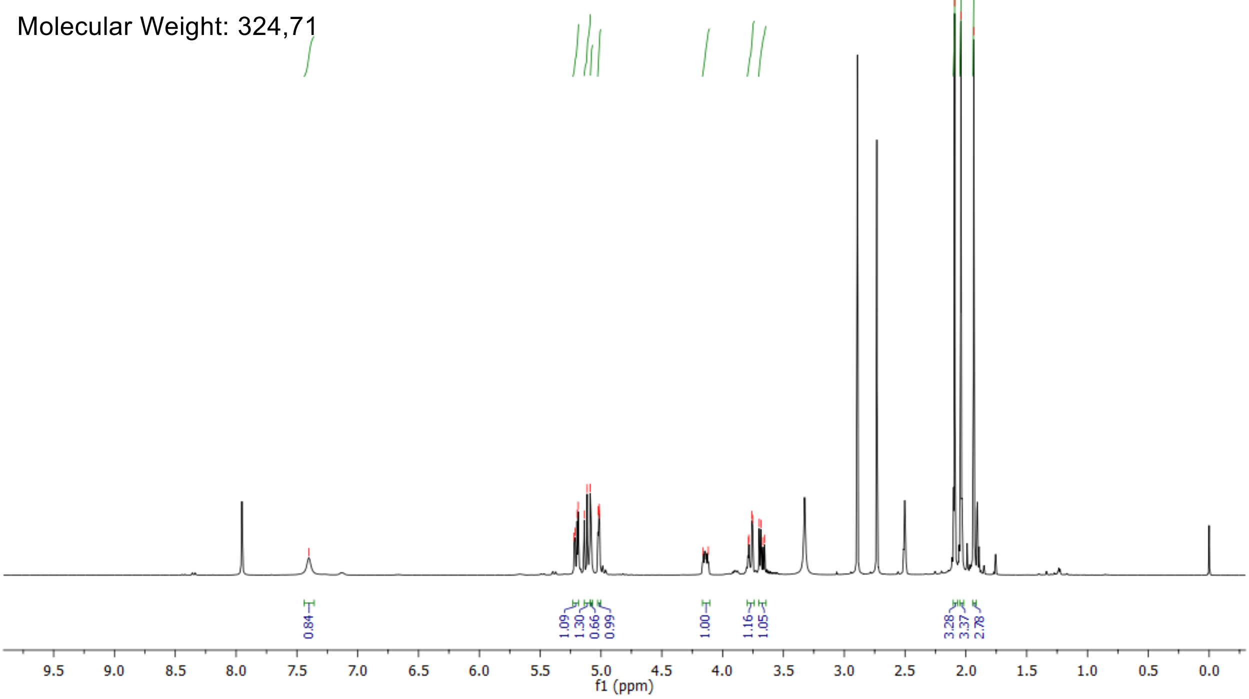

13C NMR DMSO {100 MHz}

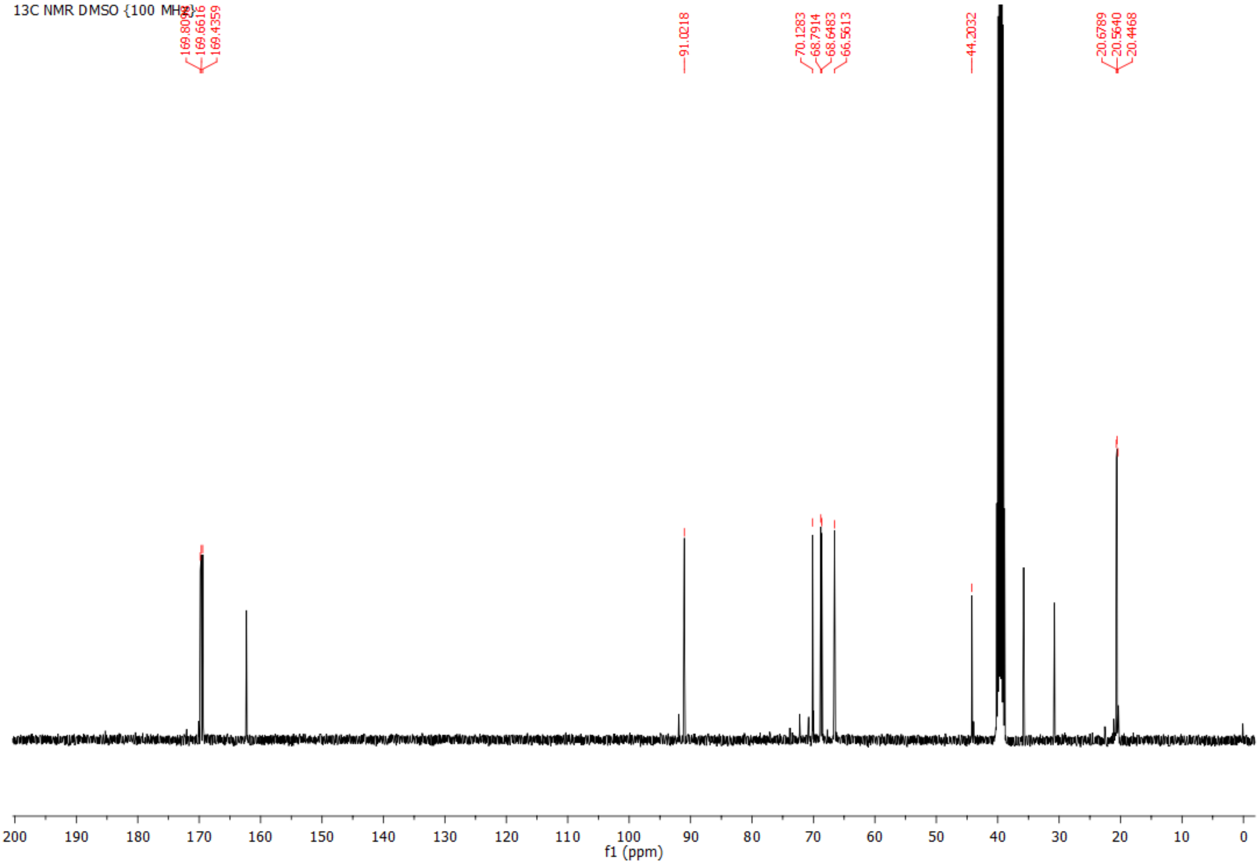

Diphenyl 6-chloro-6-deoxy-2,3,4-tri-*O*-acetyl-α-D-mannopyranosyl phosphate 16

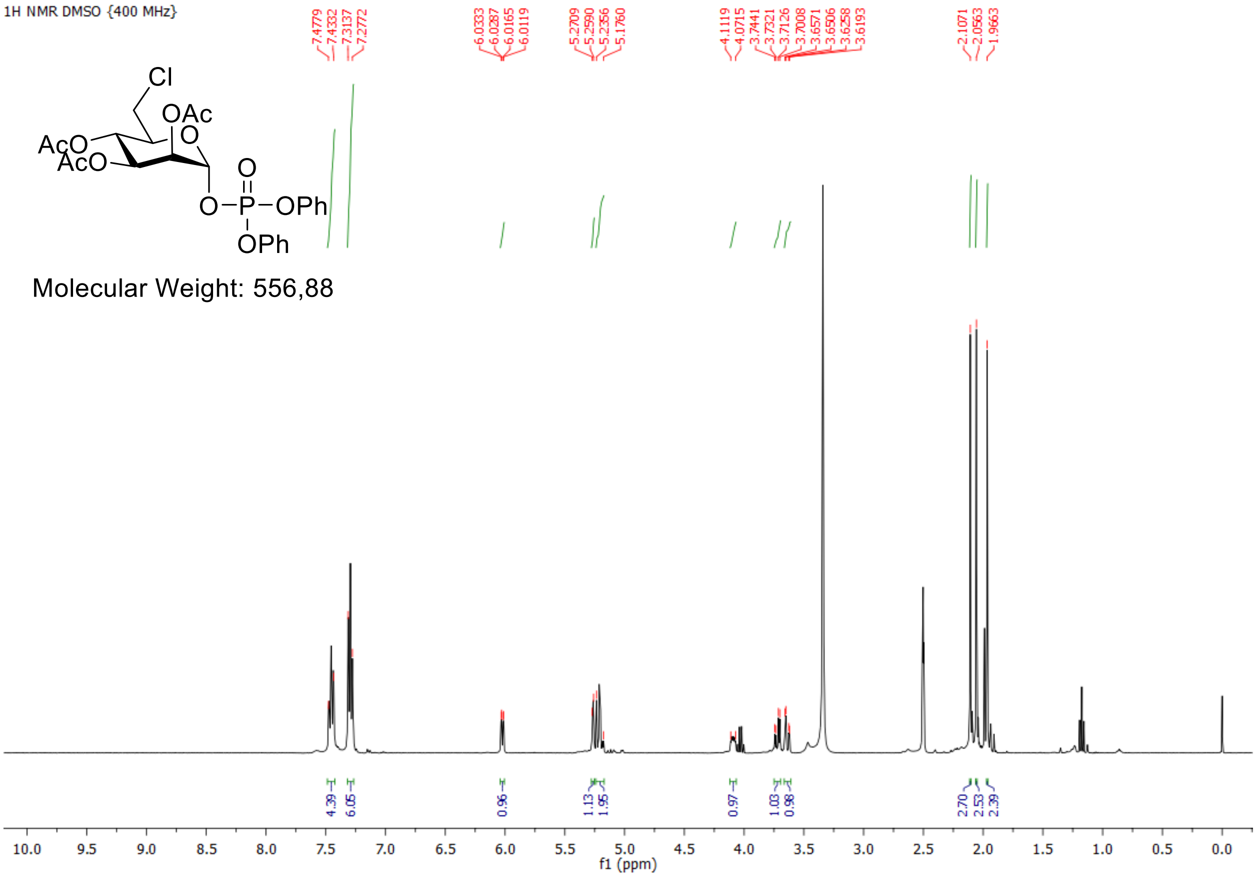

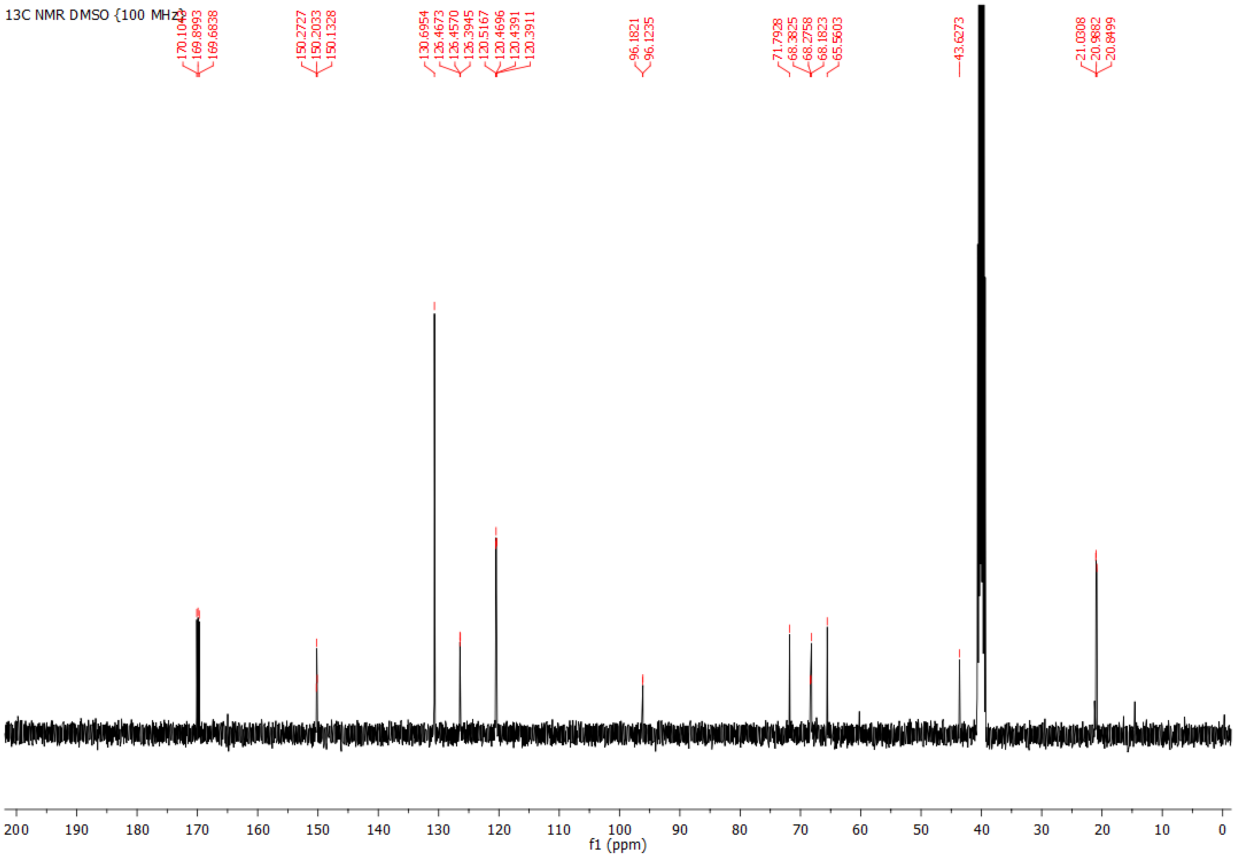

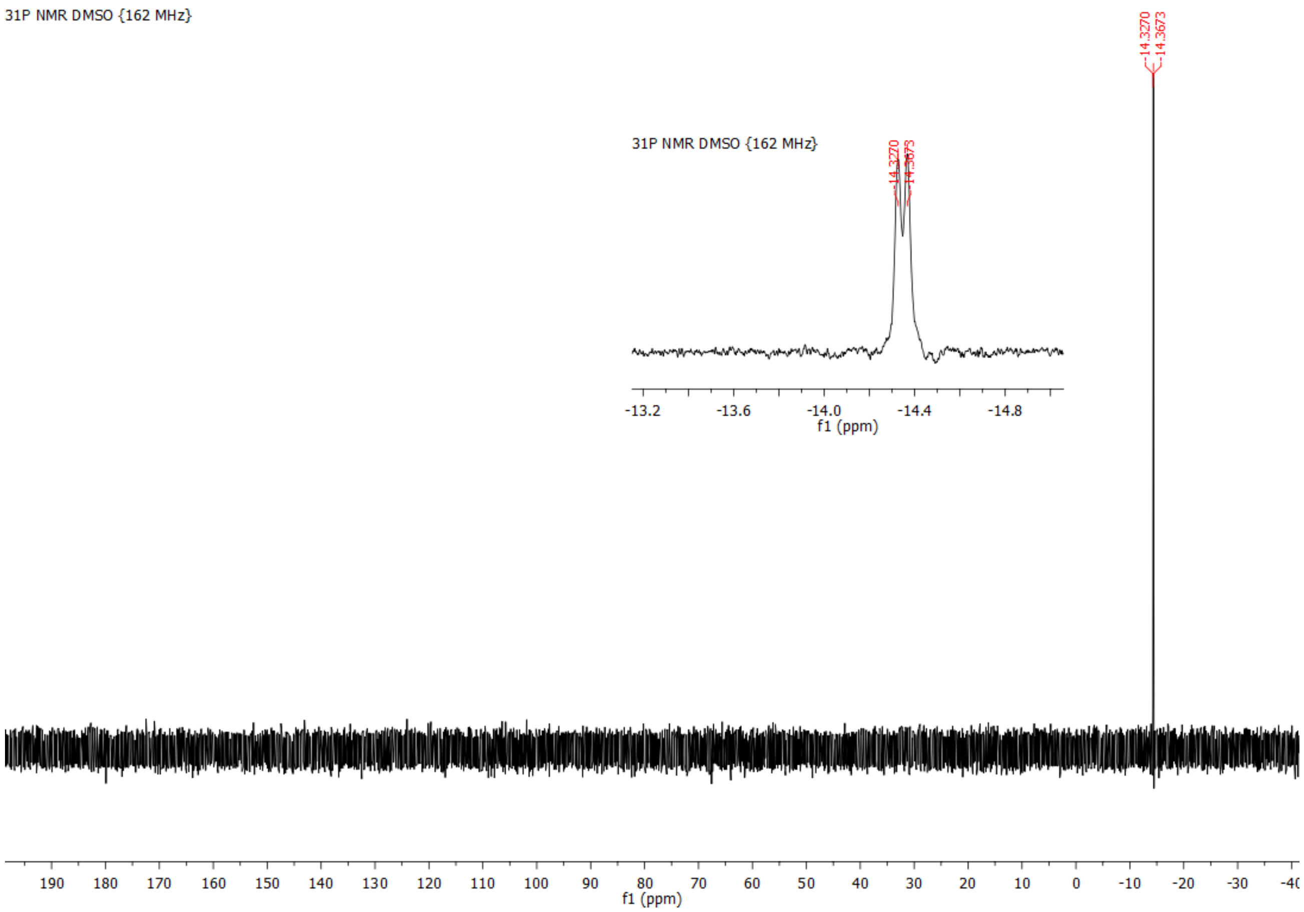
31P NMR DMSO {162 MHz}
-14.3270
-14.3673
31P NMR DMSO {162 MHz}
-14.3270
-14.3673
-13.2
-13.6
-14.0
-14.4
-14.8
f1 (ppm)
190
180
170
160
150
140
130
120
110
100
90
80
70
60
50
40
30
20
10
0
-10
-20
-30
f1 (ppm)

1,2,3-Tri-*O*-acetyl-6-chloro-6-deoxy-α-D-mannopyranose-1-phosphate

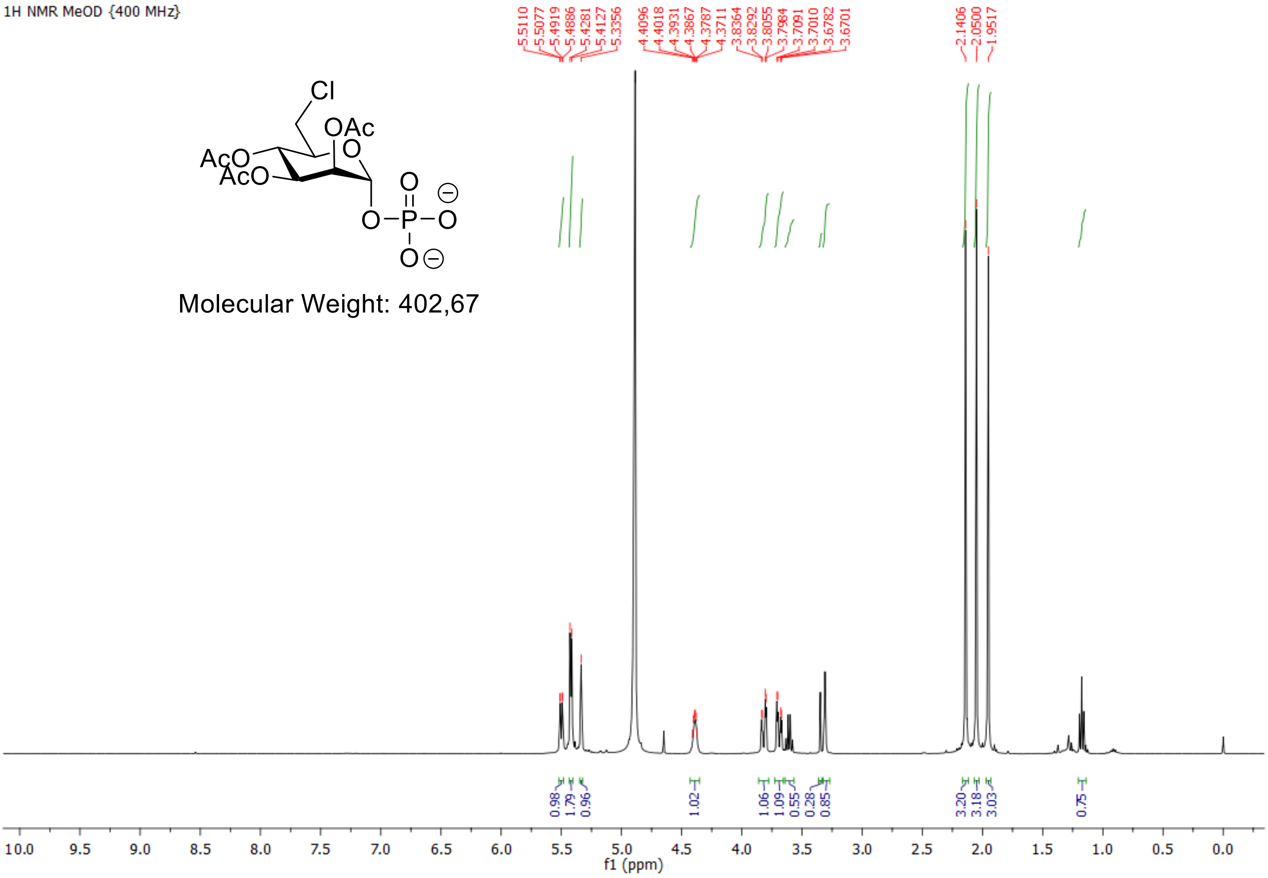

Molecular Weight: 402,67

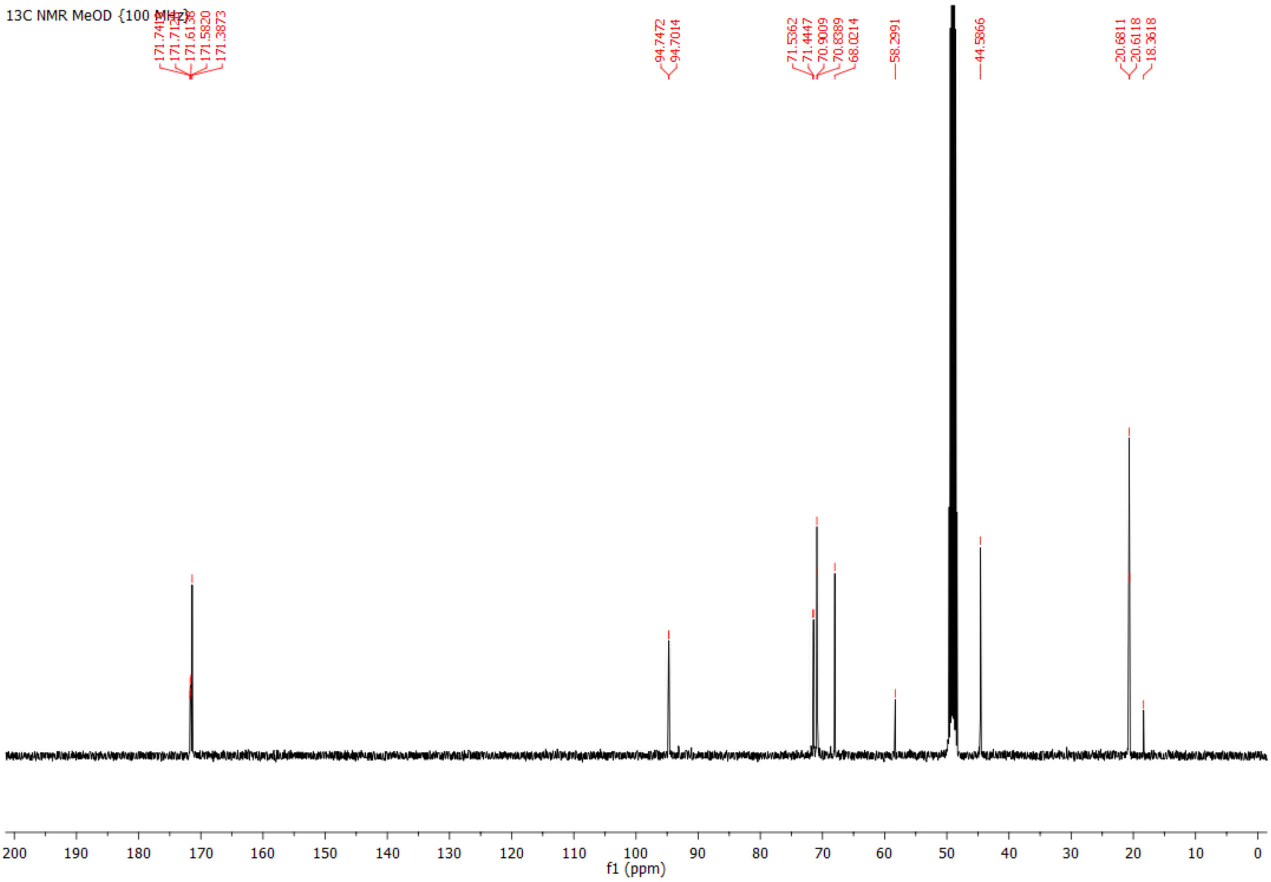

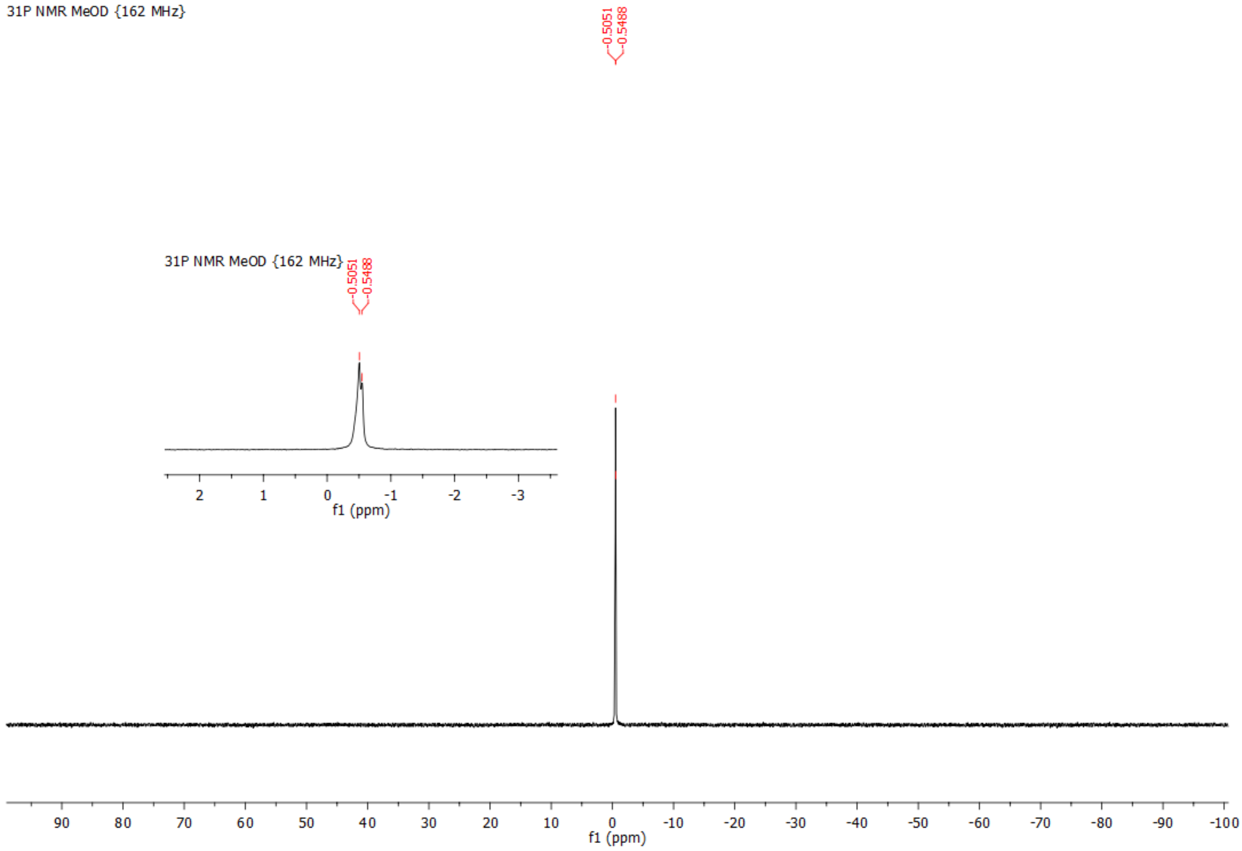
31P NMR MeOD {162 MHz}
-0.5051
-0.5488
31P NMR MeOD {162 MHz}
-0.5051
-0.5488
2
1
0
-1
-2
-3
f1 (ppm)
90
80
70
60
50
40
30
20
10
0
-10
-20
-30
-40
-50
-60
-70
-80
-90
-100
f1 (ppm)

6-Chloro-6-deoxy-α-D-mannopyranose 1-phosphate (sodium triethylamine salt) 17

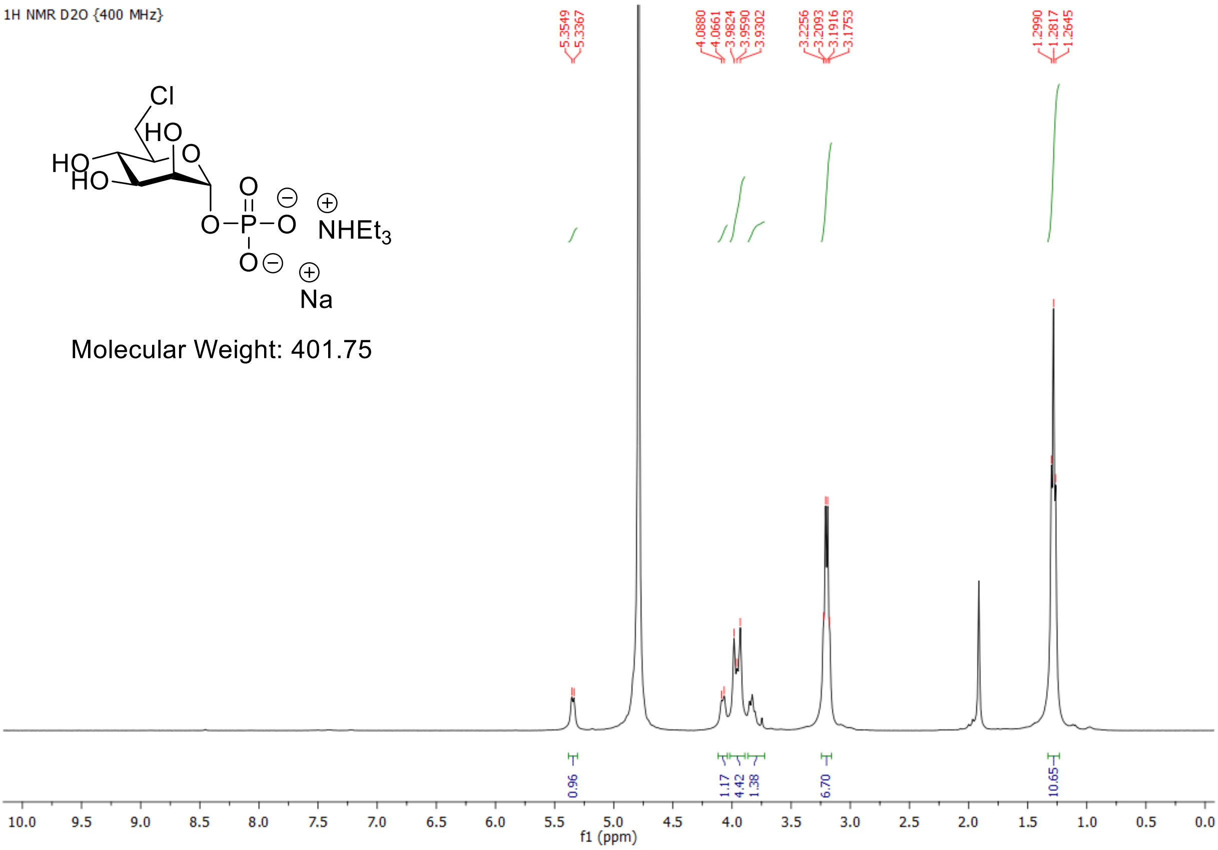

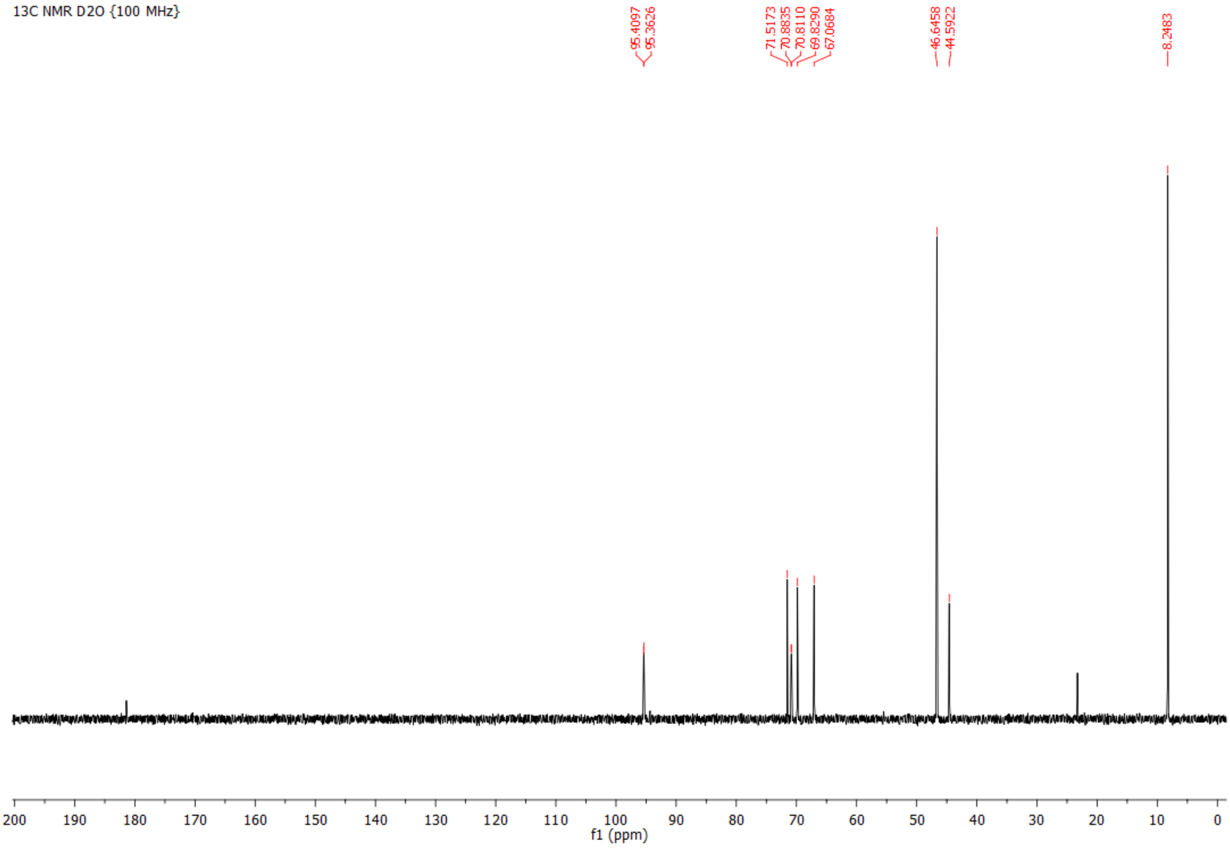

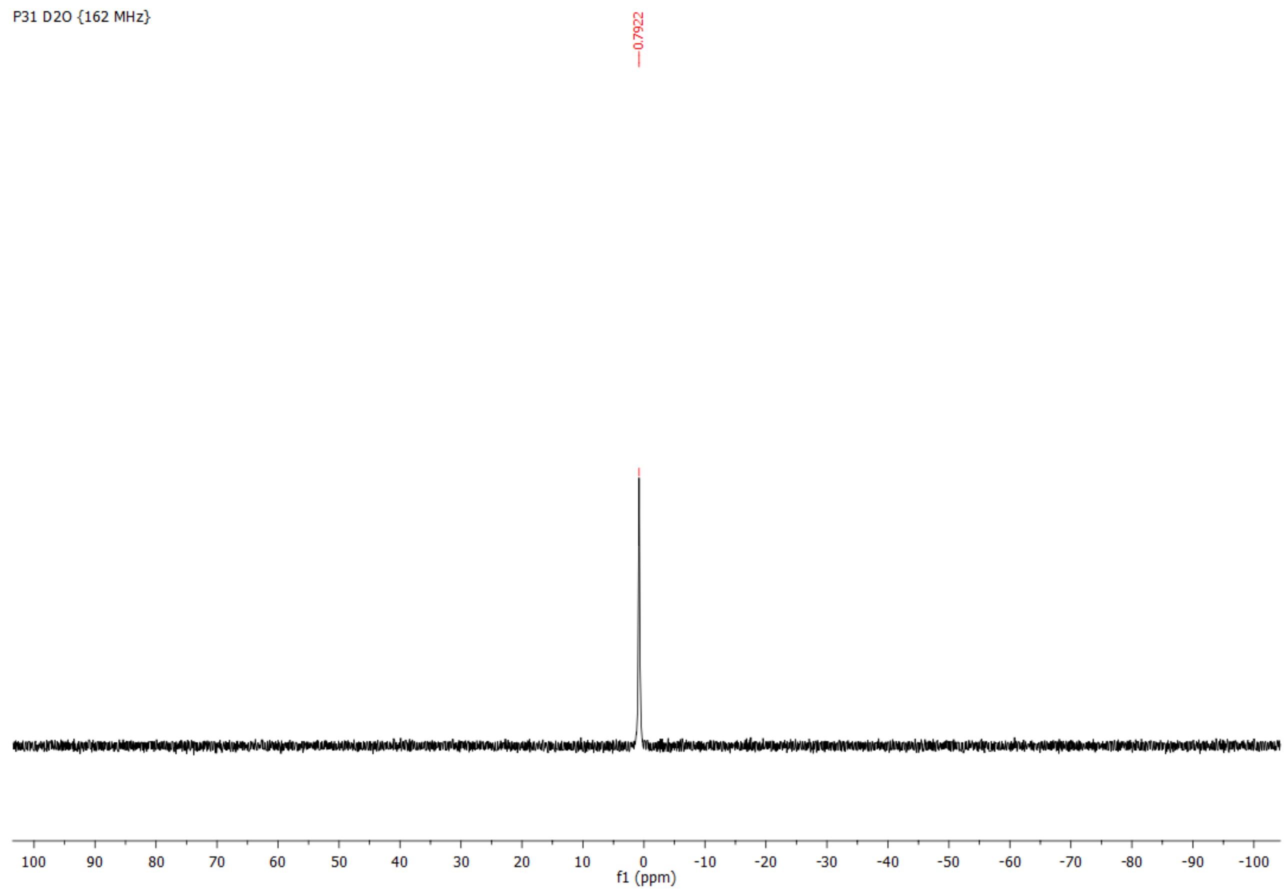
P31 D2O {162 MHz}
0.7922
100
90
80
70
60
50
40
30
20
10
0
-10
-20
-30
-40
-50
-60
-70
-80
-90
-100
f1 (ppm)

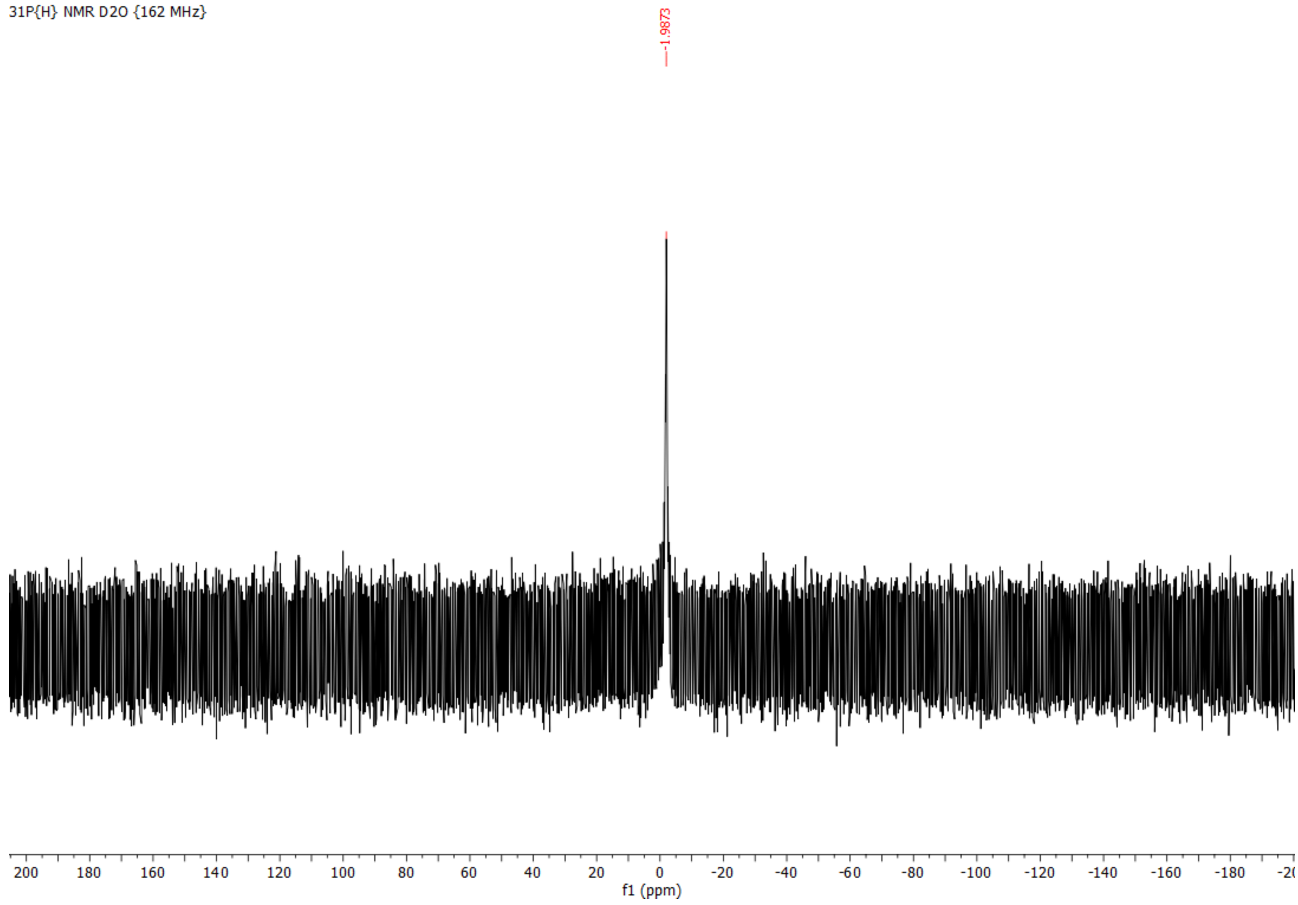
31P{H} NMR D2O {162 MHz}
-1.9873
200
180
160
140
120
100
80
60
40
20
0
-20
-40
-60
-80
-100
-120
-140
-160
-180
f1 (ppm)

Guanosine diphosphate 6-chloro-6-deoxy-α-D-mannose 19

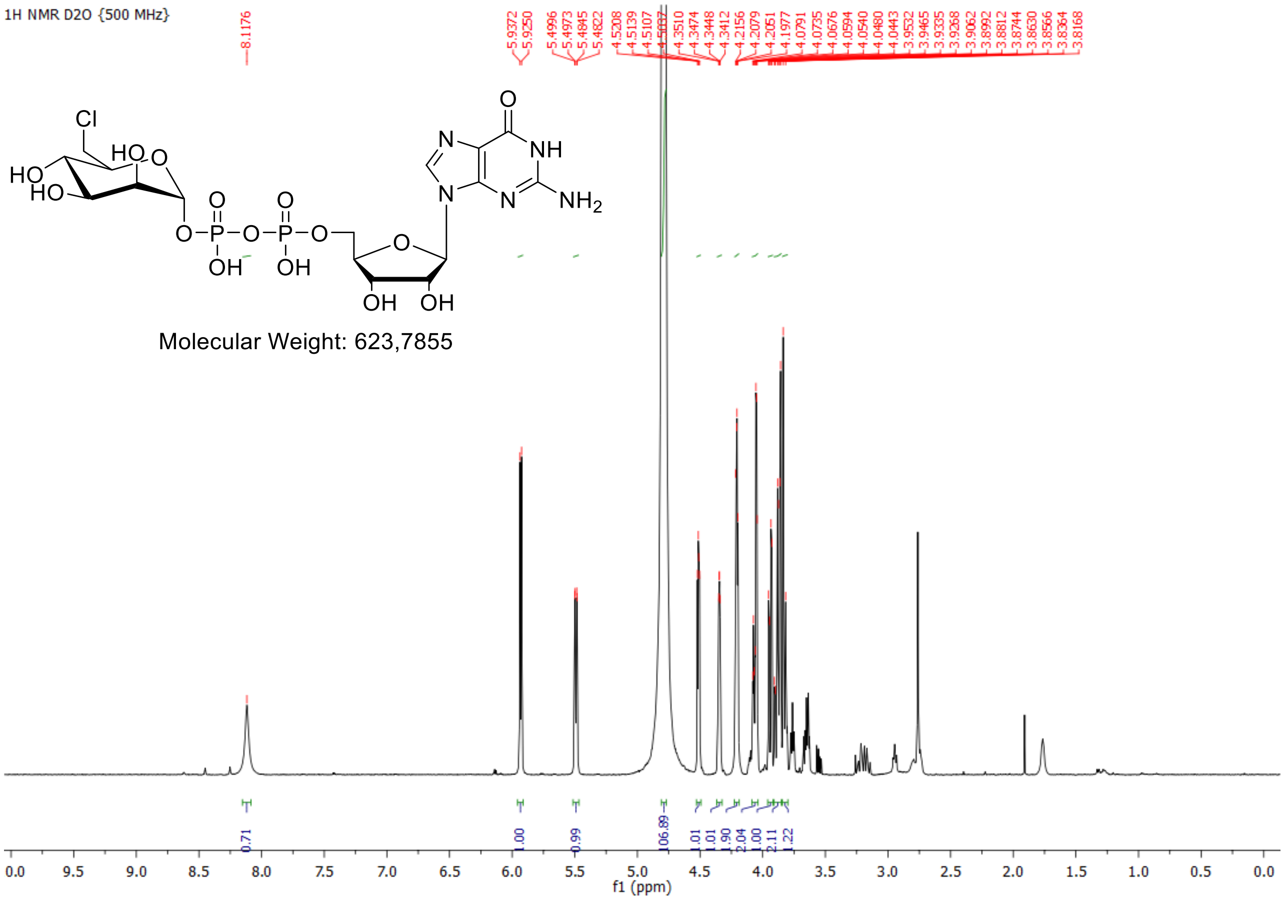

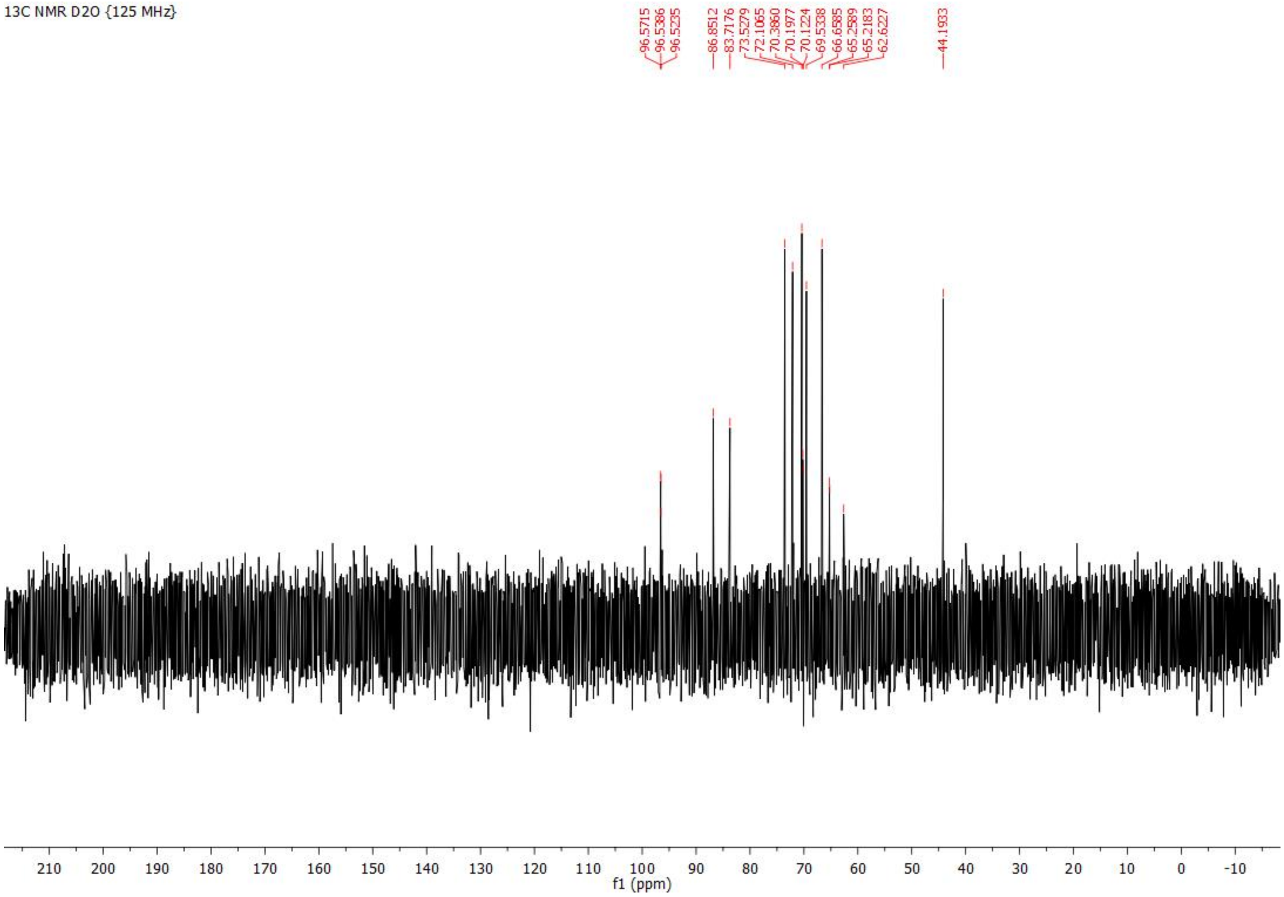

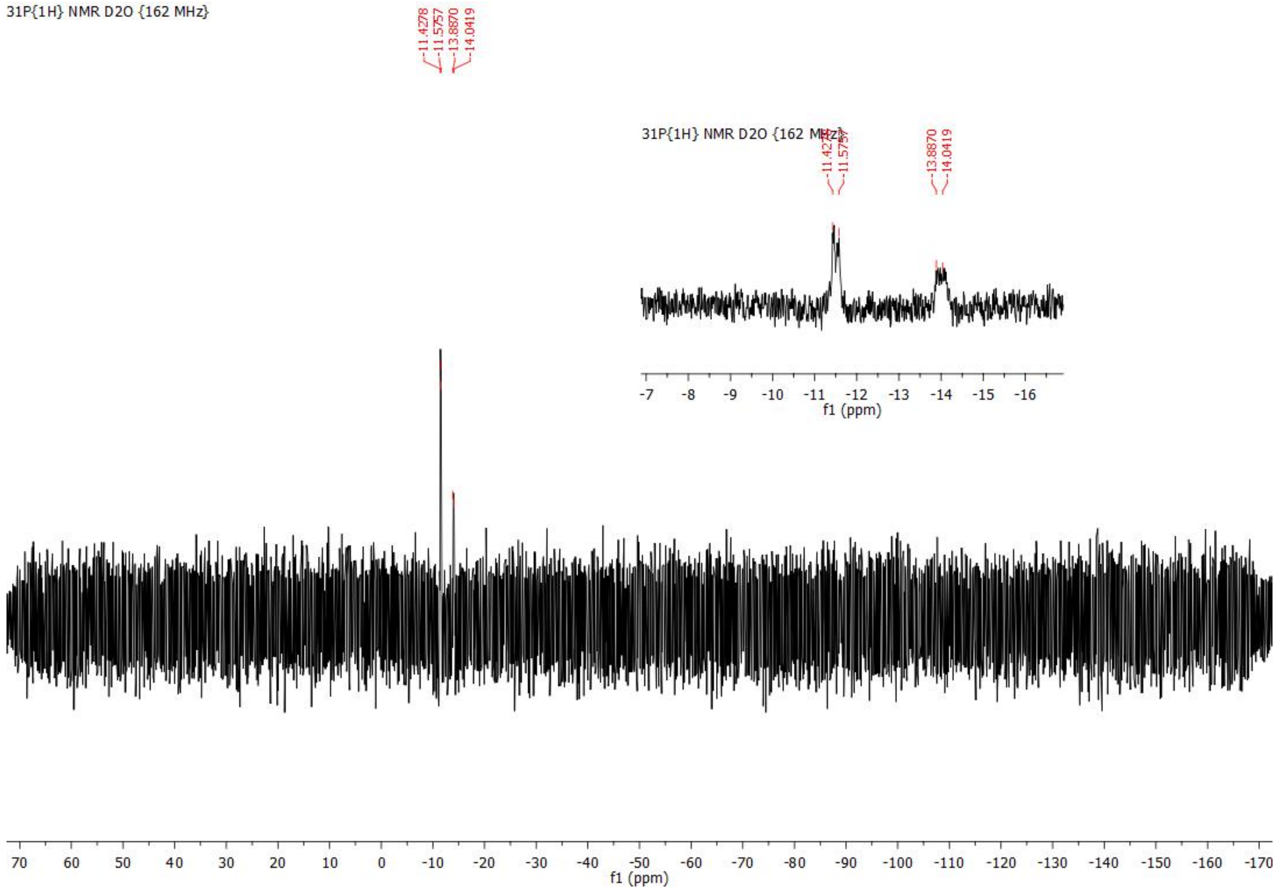

HRMS (ESI negative mode) of 6-Cl GDP-Man **19**. (^{35}Cl: M−H)$^{-}$ = 622.0335 [Δ = −4.0 ppm]; (^{37}Cl: M−H)$^{-}$ = 624.0313 [Δ = −2.8 ppm].

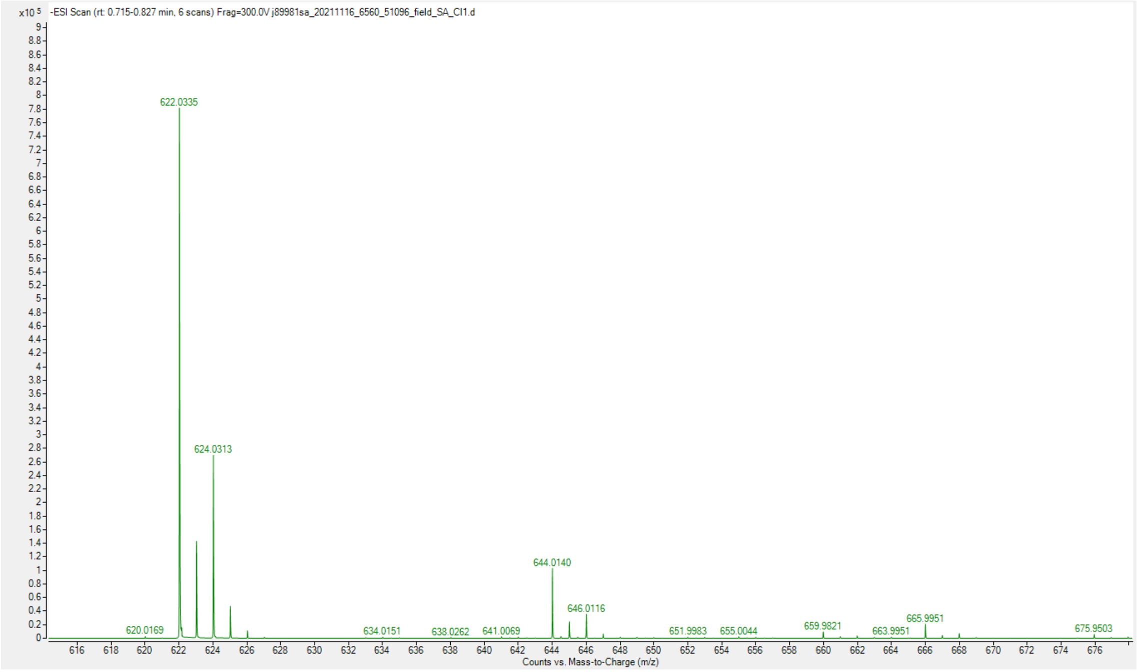

Supplement: File 1 — Detailed experimental protocols and characterisation data; spectral NMR data (1H, 13C and 31P NMR for compounds 10–17 and 19). [file Beilstein_J_Org_Chem-18-1379-s001.pdf]
